# Combined transcriptomics and proteomics unveil the impact of vitamin C in modulating specific protein abundance in the mouse liver

**DOI:** 10.1186/s40659-024-00509-x

**Published:** 2024-05-12

**Authors:** Lucie Aumailley, Antoine Bodein, Pauline Adjibade, Mickaël Leclercq, Sylvie Bourassa, Arnaud Droit, Rachid Mazroui, Michel Lebel

**Affiliations:** 1 Centre de recherche du CHU de Québec, Faculty of Medicine, Université Laval, 2705 Laurier Blvd., Local R-2714, Québec City, QC G1V 4G2 Canada; 2 Endocrinology and Nephrology Unit, CHU de Québec-Laval University Research Center, Québec City, QC Canada; 3 Cancer Research Center, Université Laval, Québec, QC G1R 3S3 Canada; 4 Proteomics Platform, Centre de recherche du CHU de Québec, Faculty of Medicine, Université Laval, Quebec City, QC G1V 4G2 Canada

**Keywords:** Vitamin C, Transcriptomics, Proteomics, Polysome profiling, Mouse liver

## Abstract

**Background:**

Vitamin C (ascorbate) is a water-soluble antioxidant and an important cofactor for various biosynthetic and regulatory enzymes. Mice can synthesize vitamin C thanks to the key enzyme gulonolactone oxidase (Gulo) unlike humans. In the current investigation, we used *Gulo*^*−/−*^ mice, which cannot synthesize their own ascorbate to determine the impact of this vitamin on both the transcriptomics and proteomics profiles in the whole liver. The study included *Gulo*^*−/−*^ mouse groups treated with either sub-optimal or optimal ascorbate concentrations in drinking water. Liver tissues of females and males were collected at the age of four months and divided for transcriptomics and proteomics analysis. Immunoblotting, quantitative RT-PCR, and polysome profiling experiments were also conducted to complement our combined omics studies.

**Results:**

Principal component analyses revealed distinctive differences in the mRNA and protein profiles as a function of sex between all the mouse cohorts. Despite such sexual dimorphism, Spearman analyses of transcriptomics data from females and males revealed correlations of hepatic ascorbate levels with transcripts encoding a wide array of biological processes involved in glucose and lipid metabolisms as well as in the acute-phase immune response. Moreover, integration of the proteomics data showed that ascorbate modulates the abundance of various enzymes involved in lipid, xenobiotic, organic acid, acetyl-CoA, and steroid metabolism mainly at the transcriptional level, especially in females. However, several proteins of the mitochondrial complex III significantly correlated with ascorbate concentrations in both males and females unlike their corresponding transcripts. Finally, poly(ribo)some profiling did not reveal significant enrichment difference for these mitochondrial complex III mRNAs between *Gulo*^*−/−*^ mice treated with sub-optimal and optimal ascorbate levels.

**Conclusions:**

Thus, the abundance of several subunits of the mitochondrial complex III are regulated by ascorbate at the post-transcriptional levels. Our extensive omics analyses provide a novel resource of altered gene expression patterns at the transcriptional and post-transcriptional levels under ascorbate deficiency.

**Supplementary Information:**

The online version contains supplementary material available at 10.1186/s40659-024-00509-x.

## Background

Maintaining adequate vitamin C (ascorbate) levels in tissues is essential for optimal body function [1]. Most mammals produce their own ascorbate in their liver and do not develop vitamin C deficiency or scurvy [2]. Humans, however, carry a mutation in the gene encoding the enzyme gulonolactone oxidase (GULO) necessary for the last step of ascorbic acid synthesis [2]. For this reason, humans depend entirely on dietary sources to obtain sufficient quantities of this vitamin. Although scurvy is nowadays rare, epidemiological studies indicate that large subpopulations (between 5% and 30% depending on age, smoking habits, or socioeconomic status) can be diagnosed with hypovitaminosis C [3–7]. Importantly, several chronic diseases and their associated risk factors, including diabetes [8], metabolic syndrome [9, 10], blood pressure [11], cardiovascular diseases [12], and all-cause mortality [13] have been inversely linked to low blood ascorbate levels.

Vitamin C has many established functions in the cells in addition to scavenging reactive oxygen species. It is a cofactor for several enzymes involved in the biosynthesis of catecholamine neurotransmitters and hormones (epinephrine, norepinephrine, and dopamine), the synthesis of carnitine essential for mitochondrial fatty acid catabolism, and the modifications of mature collagen molecules at the post-translational level [14, 15]. Such enzymes like hydroxylases or dioxygenases need Fe^2+^, 2-oxoglutarate, oxygen, and ascorbate for catalytic activity [14]. Ascorbate also controls the transcription of various genes by regulating the stability of transcription factors such as the hypoxia inducible factor 1 [16] or by modulating the epigenetic process through the regulation of the catalytic activity of several histone or DNA demethylases [14, 17, 18]. Although the influence of ascorbate on the transcriptome has been studied in several biological systems [19–21], few studies have incorporated proteomic data. This is an important facet as the protein level of a gene may change independently of its transcription rate in a tissue [22].

Clinically relevant animal models of vitamin C deficiency are essential for improving our understanding of the role of ascorbate in the pathogenesis of complex diseases [23]. Hence, a mouse knockout model lacking the enzyme gulonolactone oxidase (*Gulo*^*−/−*^) was created [24]. Ascorbate supplementation is essential to maintain viability in these mice [25]. Importantly, serum ascorbate levels in *Gulo*^*−/−*^ mice can be controlled in a non-invasive way by simply changing the concentration of ascorbate in drinking water [25, 26]. Supplementation of ascorbate in *Gulo*^*−/−*^ mice to optimal levels (to attain 40–80 µM in serum) improved their lipid profiles, mitigated aberrant serum levels of several cardiovascular risk factors, and significantly prolonged their life span [25–27]. In this context, the liver plays a fundamental role in lipid, glucose, and xenobiotic metabolisms. Indeed, it was observed that *Gulo*^*−/−*^ males will exhibit morphological changes of their hepatic mitochondria and an activation of several endoplasmic reticulum associated stress markers as a consequence of an ascorbate deficiency [25]. Note that a stressed endoplasmic reticulum and dysfunctional mitochondria in the liver can result in considerable impairments of several metabolic and detoxification hepatic activities [28]. Although there are potential sex differences associated with the hepatic redox control of mitochondrial functions [29, 30], a recent proteomic study on hepatic microsomal enriched extracts revealed that an ascorbate deficiency in *Gulo*^*−/−*^ mice specifically decreased the levels of several proteins in the mitochondrial complex III of the electron transport chain in the liver tissue without affecting the levels of other mitochondrial protein complexes [31]. However, it is unclear whether ascorbate regulates the abundance of these proteins at the transcriptional or post-transcriptional level. In the present study, we applied integrative omics analysis including transcriptomics and proteomics on the whole liver (without selectively enriching for specific hepatic subcellular compartments) to unveil the transcripts and proteins as well as the biological processes differently regulated upon ascorbate deficiency in both female and male *Gulo*^*−/−*^ mice. In addition, we conducted polysome profiling to get a better insight into the expression alterations of specific genes, potentially at the level of translation. Overall, our results indicated that ascorbate regulates the abundance of several mitochondrial complex III proteins at the post-transcriptional level in both females and males.

## Methods

### Liver harvesting from our different mouse cohorts

At the age of four months, all mice were fasted overnight before liver collection. At 10:00 am the next day, the liver was collected after final exsanguination under general anesthesia with 3% isoflurane. Liver samples were kept frozen at -80 °C.

### Experimental design and statistical rationale

**Fig. 1:**
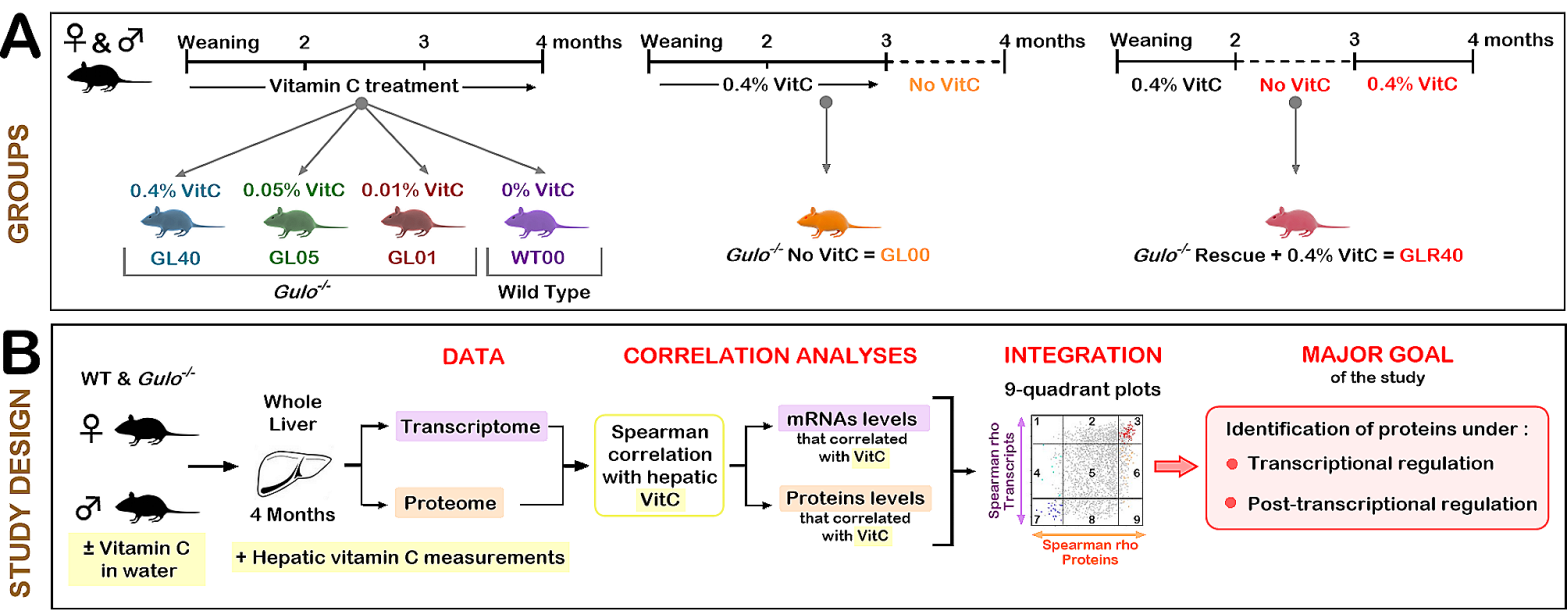
Study design and hepatic ascorbate levels in the different experimental groups of mice. (**A**) Schematic of the different vitamin C (ascorbate) treatments of mice and timeline. Females and males were separated into six experimental groups. Animals were labeled according to their genotype (GL for *Gulo*^*−/−*^ mice and WT for wild-type mice) and the vitamin C (VitC) treatments (% is weight of ascorbate per 100 mL of drinking water). The timelines for each treatment are indicated at the top. (**B**) Overview of the research design depicting the major goals of the study. Liver was harvested at the age of 4 months for all mice female and male cohorts. Transcriptomics and proteomics were performed on liver samples as well as measurements of hepatic vitamin C. Spearman rank correlation analysis of the omics data led us to the identification of proteins that are transcriptionally or post-transcriptionally regulated by ascorbate

Mice were separated into six cohorts containing at least three males and three females each as described in a previous study [31]. A schematic of the experimental groups is shown in Fig. 1A. Briefly, three cohorts of *Gulo*^*−/−*^ mice were maintained on standard diet and supplemented with 0.4%, 0.05%, or 0.01% ascorbate in drinking water from weaning until the age of four months and are referred as GL40, GL05, and GL01 mice, respectively. A fourth cohort of *Gulo*^*−/−*^ mice was supplemented with 0.4% ascorbate until the age of three months, after which ascorbate was completely removed for four weeks (GL00). A fifth cohort of *Gulo*^*−/−*^ mice was supplemented from weaning until the age of two months with 0.4% ascorbate. Ascorbate was removed from drinking water for one month and then ascorbate was reintroduced (0.4%) into drinking water until the age of four months (GLR40; referred as the rescue cohort). A wild type reference cohort (*Gulo*^*+/+*^) was maintained in the same room with no ascorbate supplementation (WT00). Total RNAs and proteins were extracted from the whole liver of each mouse for both RNA-seq analysis and label-free Liquid Chromatography-Tandem Mass Spectrometry global quantitative proteomics. Statistical criteria for each analysis in this study are detailed in the following experimental procedures sections.

### Ascorbic acid measurements in whole liver samples

Ascorbate measurements in the whole liver samples were performed by the mass spectrometry platform at TransBioTech in the city of Lévis (PQ, Canada). We described the original quantification method on an ACQUITY™ UPLC system coupled with an ACQUITY™ triple quadrupole tandem mass spectrometer Xevo-TQD (Waters, Milford, MA, USA) in the study by Aumailley et al. [31].

### Total RNA extraction

Liver tissues were homogenized in Qiazol buffer (Qiagen, Germantown, MD, USA) and hepatic total RNA was extracted using the miRNeasy Mini kit (Qiagen, Hilden, Germany) following the manufacturer’s instructions. Quantity of hepatic total RNA was measured using a NanoDrop ND-1000 Spectrophotometer (NanoDrop Technologies, Wilmington, DE, USA) and quality was assayed on an Agilent BioAnalyzer 2100 (Agilent Technologies, Santa Clara, CA, USA). Total RNA samples were frozen at -80 °C until further analysis.

### Transcriptome sequencing

The NEBNext Ultra II directional RNA library prep kit for Illumina (New Englands Biolabs Inc., Ipswich, MA, USA) was used to prepare cDNA sequencing libraries, according to the manufacturer’s instruction. Briefly, 1 µg of total RNA were purified using the NEBNext poly(A) (New Englands Biolabs Inc.) and used as a template for cDNA synthesis by reverse transcriptase with random primers. The specificity of the strand was obtained by replacing the dTTP with the dUTP. This cDNA was subsequently converted to double-stranded DNA that was end-repaired. Ligation of adaptors was followed by a purification step with AxyPrep Mag PCR Clean-up kit (Axygen, Big Flats, NY, USA), by an excision of the strands containing the dUTPs, and finally, by a PCR enrichment step of nine cycles to incorporate specific indexed adapters for the multiplexing. The quality of the final amplified libraries was examined with a DNA screen tape D1000 on a Tapestation 2200 and the quantification was done on the QuBit 3.0 fluorometer (ThermoFisher Scientific, Canada). Subsequently, RNA-seq libraries with unique index were pooled together in equimolar ratio and sequenced for paired-end 100 bp sequencing on a NovaSeq 6000 at the Next-Generation Sequencing Platform, Genomics Center, CHU de Québec Research Center-Université Laval (Québec City, PQ, Canada). The average insert size for the libraries was 290 bp. The mean number of paired reads per sample was 30 million.

### RNA-seq analysis

After sequencing, raw data were obtained in the fastq format and reads were trimmed using fastp v0.20.0 [32]. The software FastQC v0.11.8 [33] and MultiQC v1.8 [34] were used for validating the quality of the data. The alignment of the trimmed sequences to the *Mus musculus* transcriptome (Mm.Ensembl104) was performed with Kallisto v0.46.2 [35]. Only transcripts with a standard deviation of TPM (Transcripts Per kilobase Million) expression greater than zero were retained for downstream analysis. The principal component analysis (PCA) was completed with the mixOmics v6.20.0 [36] R package. The PCA graphical representations were produced with the ggplot2 v3.3.3 package [37]. Student *t*-test was performed between male and female TPMs, *p*-values were adjusted for multiple testing correction with Benjamini–Hochberg correction (false discovery rate = 0.05). Spearman rank correlation coefficient was computed for each transcript between TPM and hepatic vitamin C levels (ng/mg of tissue) across all samples. A correlation between the transcript abundance and the ascorbate concentration was considered significant if the *p*-value < 0.05 for the Spearman correlation coefficient *rho*. Only transcripts with an absolute ratio between GL40/GL00 group > 2 and at least 13 quantified values among the 18 female or male samples (thus > 70% of the total samples quantifiable for females or males) were considered for the correlation analysis. Student *t*-test and Spearman rank correlations were done in R v4.2.1.

### Isolation of polysomal associated RNAs and polysomal profiles analysis

Isolation of polysomal associated RNAs and polysomal profiles analysis were performed like previously described [38]. Briefly, livers from male *Gulo*^*−/−*^ mice treated with 0% or 0.4% ascorbate were homogenized on ice by 10 strokes in an ice-cold Dounce homogenizer with 2 ml of lysis buffer (20 mM Tris, pH 7.4, 150 mM NaCl, 5 mM MgCl2, 40 U/mL Rnasin, 50 µg/mL cycloheximide, cOmplete™ EDTA-free Protease Inhibitor Cocktail (Roche, Indianapolis, IN, USA) and 1 mM dithiothreitol), and Nonidet P-40 substitute and sodium deoxycholate was added to a final concentration of 1% for lysis. Extracts were kept 15 min on ice and then clarified by centrifugation at 12,000 *g* for 20 min at 4 °C. RNA concentration was measured by spectrophotometry and ∼20 OD260 units of RNA were overlaid on top of a 15–55% sucrose gradient. The gradients were centrifuged at 223,000 *g* (SW 40 TI Beckman rotor, Indianapolis, IN, USA) for 2.5 h. Fractions were collected into individual tubes with continuous monitoring of absorbance at 254 nm in an Automated Density Fractionation System (Teledyne Isco, Density Gradient Fractionation System, LabWrench, Midland, ON, Canada). To generate polysomal charts, absorbance was recorded on a chart paper. RNA from each fraction was extracted by phenol–chloroform extraction and fractions corresponding to monosomes, light, or heavy polysomes (1 and 2) were respectively pooled together.

For quantification of the area under the curves in the polysome graphs, polysome profiles from four biological replicates for ascorbate-deficient *Gulo*^*−/−*^ mice (GL00), or five biological replicates for *Gulo*^*−/−*^ mice treated with 0.4% vitamin C (GL40) were scanned and digitized. The number of pixels for sub-polysomal fractions were calculated using ImageJ to obtain the area under the curve (i.e. area corresponding to peaks of the 40 S, 60 S and 80 S ribosomal sub-units) and for polysomal fractions (i.e. area from the first polysomal peak to the twelfth polysomal peak, as reported [38]). The values for the lower limit of the Y axis were defined by the lowest points on each graph. Results are presented as polysomal fractions area relative to total fractions area for each condition. Significant difference between GL00 and GL40 was determined via a non-parametric t-test.

### Quantitative real-time PCR

Quantitative Real-Time PCR measurements were performed by the CHU de Québec Research Center (CHUL) Gene Expression Platform (Québec, PQ, Canada) and were compliant with MIQE guidelines [39, 40]. Briefly, first-strand cDNA synthesis was accomplished using 3 µg of isolated RNA in a reaction containing 200 U of Superscript IV Rnase H-RT (Invitrogen Life Technologies, Burlington, ON, Canada), 300 ng of oligo-dT_18_, 50 ng of random hexamers, 50 mM Tris-HCl pH 8.3, 75 mM KCl, 3 mM MgCl_2_, 500 µM deoxynucleotides triphosphate, 5 mM dithiothreitol, and 40 U of Protector Rnase inhibitor (Roche Diagnostics, Indianapolis, IN, USA) in a final volume of 50 µL. Reaction was incubated at 25 °C for 10 min, then at 50 °C for 20 min, and inactivated at 80 °C for 10 min. Complementary DNA was purified with a PCR purification kit (Qiagen, Hilden, Germany).

Oligoprimer pairs (synthesized by Integrated DNA Technology, Coralville, IA, USA) were designed by GeneTool 2.0 software (Biotools Inc., Edmonton, AB, Canada) and their specificity was verified by blast in the GenBank database. Complementary DNA corresponding to 0.4 or 20 ng of total RNA was used to carry out fluorescent based Realtime PCR quantification using the LightCycler 480 (Roche Diagnostics, Mannheim, Germany). Reagent LightCycler 480 SYBRGreen I Master (Roche Diagnostics, Indianapolis, IN, USA) was used as described by the manufacturer. The conditions for PCR reactions were: 45 cycles, denaturation at 98 °C for 10 s, annealing at 60 °C for 10 s, elongation at 72 °C for 14 s and then 74 °C for 5 s (reading). Relative quantity was calculated using second derivative method and by applying the delta Ct method [41]. For total RNA analysis, normalization was performed using the reference genes known to have stable expression levels from embryonic life through adulthood in various tissues [42]: ATP synthase, H + transporting, mitochondrial F1 complex, O subunit (Atp5o) and ubiquitin C (Ubc). Normalization with 18 S rRNA was used for ribosome associated RNAs analysis as described before [43].

### Whole liver protein extraction and preparation for label-free Liquid Chromatography-Tandem mass spectrometry

Preparation of protein samples for mass spectrometry analysis were performed as described previously in the study by Aumailley et al. [31]. Briefly, tissue protein extraction was carried out in lysis buffer containing 50 mM Tris-HCl (pH 7.5), 1% NP-40, 0.2% SDS, 1% sodium deoxycholate, 150 mM NaCl, 1mM PMSF, complete protease inhibitor cocktail and phosphatase inhibitor cocktail PhoSTOP™ (Roche Applied Science, Indianapolis, IN, USA). After tissue homogenization and sonication, samples were centrifuged at 16,000 *g* for 15 min thrice. Five volumes of acetone were added to the lysate to allow the proteins to precipitate overnight. The protein pellet was recovered by centrifugation at 16,000 *g* for 15 min and resuspended in 200 µL of 50 mM ammonium bicarbonate / 1% DOC buffer. Protein concentration of each sample was measured by the Bradford protein assay (Bio-Rad, Mississauga, ON, Canada).

Ten µg of proteins were reduced with 0.2 mM dithiothreitol (DTT) for 30 min at 37 °C and alkylated with 0.8 mM iodoacetamide for 30 min at 37 °C. Samples were then incubated with trypsin (trypsin: protein ; 1:50) at 37 °C overnight. The reaction was stopped by addition of 1% trifluoroacetic acid (TFA), 0.5% acetic acid, and 0.5% acetonitrile then centrifuged for 5 min at 16,000 *g*. The peptides obtained were then desalted using C18 stagetip.

### Label-free Liquid Chromatography-Tandem mass spectrometry analysis

One µg of each liver sample was analyzed by nanoLC/MSMS using a Dionex UltiMate 3000 nanoRSLC chromatography system (Thermo Fisher Scientific) connected to an Orbitrap Fusion mass spectrometer (Thermo Fisher Scientific) equipped with a nanoelectrospray ion source described previously [26, 31]. Peptides were trapped at 20 µL/min in loading solvent (2% acetonitrile, 0.05% TFA) on a 5 mm x 300 μm C18 pepmap cartridge pre-column (Thermo Fisher Scientific) for 5 min. Then, the pre-column was switched online with Pepmap Acclaim column (Thermo Fisher Scientific) 50 cm x 75 μm internal diameter separation column and the peptides were eluted with a linear gradient from 5 to 40% solvent B (A: 0.1% formic acid, B: 80% acetonitrile, 0.1% formic acid) in 90 min, at 300 nL/min for a total run time of 120 min. Using the Thermo Xcalibur software version 4.1.50, mass spectra were acquired with the data dependent acquisition mode. Full scan mass spectra (350 to 1800 m/z) were acquired in the orbitrap using an AGC target of 4e5, a maximum injection time of 50 ms, and a resolution of 120,000. Internal calibration using lock mass on the m/z 445.12003 siloxane ion was used. Each MS scan was followed by acquisition of fragmentation MS/MS spectra of the most intense ions for a total cycle time of 3 s (top speed mode). The selected ions were isolated using the quadrupole analyzer in a window of 1.6 m/z and fragmented by Higher energy Collision-induced Dissociation with 35% of collision energy. The resulting fragments were detected by the linear ion trap in rapid scan rate with an AGC target of 1e4 and a maximum injection time of 50 ms. Dynamic exclusion of previously fragmented peptides was set for a period of 30 s and a tolerance of 10 ppm.

### Database searching and label free quantification (LFQ)

Spectra were searched against the Uniprot Ref *Mus musculus* database (July 2020 release/ 63,807 entries) using the Andromeda module of MaxQuant software v. 1.6.10.43 [44]. Trypsin/P enzyme parameter was selected with two possible missed cleavages. Carbamidomethylation of cysteins was set as fixed modification while methionine oxidation, protein N-terminal acetylation and hydroxyproline were set as variable modifications for the global search. Mass search tolerance was 5 ppm and 0.5 Da for MS and MS/MS, respectively. For protein validation, a maximum False Discovery Rate of 1% at peptide and protein level was used based on a target/decoy search. MaxQuant was also used for Label Free Quantification. The ‘match between runs’ option was used with 20 min value as alignment time window and 0.7 min as match time window. Only unique and razor peptides were used for quantification. Normalisation (LFQ intensities) was performed by MaxQuant.

### LFQ data post-processing and statistical analysis

Post-processing of the LFQ data were performed like previously described [26, 31]. Briefly, Rstudio 1.2.5019 was used for data post-processing. In the case of protein intensity values that were missing, values were replaced by a noise value corresponding to 1% percentile of the normalised value for each condition. A protein was considered as quantifiable only if at least three intensity values in the three replicates of one of the two conditions being compared were present and if two peptides or more were identified for this protein. Multi-scatter plots were generated with Perseus software v2.0.7.0 [45] using the log2 transformed LFQ intensities. Pearson correlation coefficients between the different triplicated within the various treatment groups were calculated. The principal component analysis (PCA) was completed with the mixOmics v6.20.0 [36] R package. The PCA graphical representations were produced with the ggplot2 v3.3.3 package [37]. Student *t*-test was performed between male and female LFQ intensities, *p*-values were adjusted for multiple testing correction with Benjamini–Hochberg correction (false discovery rate = 0.05). Spearman rank correlation coefficient was computed for each protein between LFQ intensity and hepatic vitamin C levels (ng/mg of tissue) across all samples. A correlation between the protein abundance and the ascorbate concentration was considered significant if the *p*-value < 0.05 for the Spearman correlation coefficient *rho*. Only proteins with an absolute ratio between GL40/GL00 group > 2 and at least 13 quantified values among the 18 female or male samples (thus > 70% of the total samples quantifiable for females or males) were considered for the correlation analysis. Student *t*-test and Spearman rank correlations were done in R v4.2.1.

### Combined analysis of the transcriptome and proteome Spearman correlation studies

Nine-quadrant scatter plots were created with Perseus software v2.0.7.0 [45]. Spearman’s rank correlations between transcripts or proteins levels and hepatic ascorbate levels results were used as x and y values, respectively, for each identified protein that had a corresponding transcript with quantifiable information in females or males. The dashed lines on the x and y axes were set at a *rho* value equal to 0.46876 or -0.46876. These lines indicated the significance thresholds (*p*-value < 0.05) and drawn nine distinctive quadrants on the scatter plots. Genes that exhibited at least a two-fold protein abundance difference between ascorbate-deficient *Gulo*^*−/−*^ mice and *Gulo*^*−/−*^ mice treated with 0.4% ascorbate in drinking water since weaning were colored in quadrants one, three, four, six, seven and nine.

### Functional annotation analysis

Enriched biological processes were identified using DAVID (Database for Annotation, Visualization, and Integrated Discovery) web site [46]. We also used STRING (Search Tool for the Retrieval of Interacting Genes) to generate a network of protein interactions [47].

### Immunoblotting analysis

Western blotting analyses on whole liver protein samples were performed as previously described [31]. The separated proteins on the PVDF membranes were detected using the following antibodies: mouse monoclonal antibodies against ubiquinol-cytochrome c reductase, Rieske iron-sulfur polypeptide 1 (anti-Uqcrfs1 [5A5] ab14746), ubiquinol-cytochrome C reductase core protein I (anti-Uqcrc1 [16D10AD9AH5] ab110252), ubiquinol-cytochrome C reductase core protein II (anti-Uqcrc2 [13G12AF12BB11] ab14745) from Abcam (Cambridge, MA, USA) and mouse monoclonal antibody β-actin (A5441) from Sigma-Aldrich (Oakville, ON, Canada).

## Results

### Study design and hepatic ascorbate levels in the different experimental groups of mice

To identify the biological processes that are altered in the whole hepatic tissue of mice exhibiting different degrees of vitamin C deficiency, specific cohorts of female and male mice were subjected to different ascorbate concentrations in drinking water. The Fig. 1A presents the different mouse groups (WT and *Gulo*^*−/−*^ mice) treated with the indicated concentrations of ascorbate in drinking water. Succinctly, six different cohorts containing at least three females and three males were used in this study. *Gulo*^*−/−*^ mice were treated from weaning until the age of four months with 0.4% (GL40), 0.05% (GL05) or 0.01% (GL01) ascorbate (w/v). One cohort of *Gulo*^*−/−*^ mice underwent a four-week ascorbate depletion from the age of three months until the age of four months (GL00). Another cohort of *Gulo*^*−/−*^ mice were supplemented with 0.4% ascorbate (w/v) from weaning to the age of two months after which they experienced a four-week ascorbate depletion. At the age of three months, 0.4% ascorbate (w/v) was added back (for rescue) to drinking water until the age of four months (GLR40). The wild type (WT00) control mice, which synthesize their own ascorbate, were used as the normal reference cohort without ascorbate supplementation in drinking water. Mice were fed ad libitum with normal chow and were not treated with any drugs or chemicals that can induce liver lesions. We performed both transcriptomic and proteomic analyses on the whole liver samples of all these animals at the age of four months as described in the experimental design of Fig. 1B. Importantly, using UPLC-MS/MS in a previous study, we had measured ascorbate levels in the whole hepatic tissue of the exact same animals that are being analyzed in the present transcriptomic/proteomic study (Fig. 1A) [31]. The raw data of such measurements are shown in the Additional file 1: Table S1. The major goal was to identify proteins which abundance was regulated by vitamin C at the transcriptional or post-transcriptional levels in the whole liver of these mice.

### Biological reproducibility of label-free quantification of whole liver proteomes

To determine whether the proteomic profiles differ between the different groups of mice, we performed quantitative proteomic profiling with label-free LC-MS/MS. Normalization of the data was performed on the LFQ intensities using MaxQuant. As indicated in the Additional file 2: Figure S1A, the distribution of the log2 transformed protein intensities was comparable as the data for each liver sample were homogenized and distributed at the same level. Out of the 3,414 proteins identified in all the different liver samples, 2,393 proteins were quantified with at least two peptides when we compared all the female to the male samples (Additional file 3: Table S2). Notably, the number of quantified proteins for each individual sample was similar regardless of ascorbate treatments within the female or the male groups (Additional file 2: Figure S1B). The pie chart in the Additional file 2: Figure S1C provides the protein coverage distribution for all the identified and quantified proteins. More than 30% of the data set contained proteins that were represented by identified peptides covering more than 50% of their sequence.

To examine the variation among the different biological replicates within the same experimental groups, multi-scatter plots were generated using Perseus software. As shown in the Additional file 4: Figure S2, Pearson correlation coefficients between the different triplicates within the various treatment groups (GL00, GL01, GL05, GL40, GLR40, and WT00 for females or males) varied from 0.8668 to 0.9914. These results indicated a high degree of correlation among different triplicates within each treatment groups.

### Sex-based difference in liver transcriptomic and proteomic profiles regardless of ascorbate treatments

Liver tissues from the same mice were used for both the transcriptomic and proteomic analyses.

**Fig. 2:**
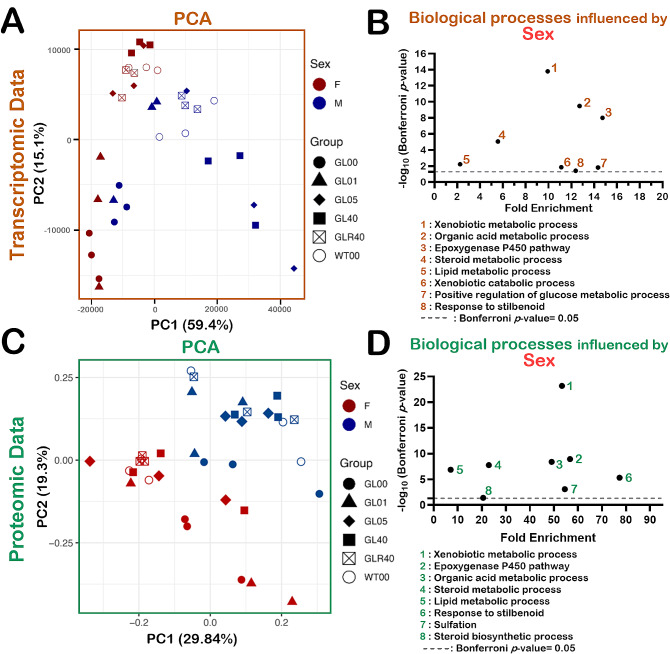
Impact of sexual dimorphism on the liver transcriptomic and proteomic profiles of mice treated with different concentrations of ascorbate. (**A**) Principal Component Analysis (PCA) on the transcriptomic data. (**B**) One-dimensional Gene Ontology analysis showing the fold enrichments of genes involved in biological processes significantly different between females and males. (**C**) PCA graph of *Gulo*^*−/−*^ mice treated with different concentrations of ascorbate in drinking water since weaning compared to age-matched control untreated WT mice in both females and males. (**D**) One-dimensional Gene Ontology analysis showing the fold enrichments of proteins involved in biological processes significantly different between females and males. For the PCA graphs, F = Females and M = Males; WT00 = WT females or males with no ascorbate for 4 months; GL00 = *Gulo*^*−/−*^ females or males with no ascorbate for 1 month; GL01 = *Gulo*^*−/−*^ females or males treated with 0.01% ascorbate for 4 months; GL05 = *Gulo*^*−/−*^ females or males treated with 0.05% ascorbate for 4 months; GL40 = *Gulo*^*−/−*^ females or males treated with 0.4% ascorbate for 4 months; GLR40 = *Gulo*^*−/−*^ females or males treated with 0% ascorbate for 1 month followed by 1 month of 0.4% ascorbate treatment; *N* = 3 for each experimental groups

Total RNA was extracted from the liver for next-generation RNA sequencing. The Additional file 5: Table S3 provides the transcripts per kilobase million or TPM value for 36,066 genes in each sample. There were 26,071 transcripts identified in at least one of the female or male samples. To determine whether the transcriptomic profiles differed between groups of ascorbate-treated mice, we first performed a Principal Component Analysis (PCA) on the transcriptomic data (Additional file 5: Table S3). As indicated in Fig. 2A, a clear distinction between males (blue symbols) and females (red symbols) transcriptomic profiles was appreciable among mice, and this regardless of the ascorbate treatment. The Additional file 6: Table S4 displays the 700 transcripts with a significant difference of more than two-fold between females and males (adjusted *p*-value < 0.05). Biological processes that were different between females and males were evaluated using the Database for Annotation, Visualization and Integration Discovery (DAVID) tool [46]. The Fig. 2B indicates that eight biological processes were significantly different between females and males with a Bonferroni *p*-value < 0.05. The lists of transcripts for each biological process are indicated in the Additional file 7: Table S5.

We next performed a PCA using LFQ normalized and imputed data from the Additional file 3: Table S2. As indicated in Fig. 2C, all the *Gulo*^*−/−*^ and wild type females (all the red symbols on the graph) showed little overlap with the *Gulo*^*−/−*^ and wild type male groups (all the blue symbols). Thus, the proteomic results also pointed out to the sex-associated difference observed in the transcriptomic profiles of our various mouse cohorts. The Additional file 8: Table S6 displays the 76 proteins with a significant difference of more than two-fold between all the females and all the males (adjusted *p*-value < 0.05). Biological processes that were different between females and males were also evaluated using the DAVID bioinformatics tool [46]. The analysis indicated that proteins exhibiting different abundance between female and male liver tissues were involved in seven different biological pathways (Fig. 2D). The Additional file 9: Table S7 provides a list of proteins corresponding to these different biological processes. Six of these pathways were commonly affected at the transcriptomic and proteomic levels (Fig. 2B and D) and included the xenobiotic metabolic process, steroid metabolic process, epoxygenase P450 pathway, organic acid metabolic process, response to stilbenoid, and lipid metabolic process.

### Liver ascorbate levels impact the hepatic transcriptomic and proteomic profiles of females and males

Further examination of the PCA in Fig. 2A indicated that the transcriptome profiles of *Gulo*^*−/−*^ females treated with 0% (closed red circles) or 0.01% ascorbate (red triangles) did not overlap with the other *Gulo*^*−/−*^ females treated with higher levels of ascorbate in drinking water or with untreated WT females. *Gulo*^*−/−*^ males treated with 0% (closed blue circles) ascorbate from the age of three to four months did not overlap with *Gulo*^*−/−*^ males treated with more than 0.05% ascorbate in drinking water. In contrast to females, two *Gulo*^*−/−*^ males treated with 0.01% ascorbate (blue triangles) were closer to groups of mice with high ascorbate concentrations in their liver (such as GLR40 and WT00) than to all the GL00 males.

Although the *Gulo*^*−/−*^ females that experienced a four-week depletion followed by a four-week 0.4% ascorbate treatment (FGLR40: open red squares) were close to *Gulo*^*−/−*^ females treated with 0.4% ascorbate since weaning (FGL40: closed red squares in Fig. 2A), they did not overlap with them. A similar result was observed for the *Gulo*^*−/−*^ males (MGLR40: open blue squares *versus* MGL40: closed blue squares). These observations indicate that the transcriptomic profiles of ascorbate-deficient *Gulo*^*−/−*^ mice were not fully re-established by a four-week treatment of 0.4% ascorbate in drinking water (rescue experiment) compared to *Gulo*^*−/−*^ mice treated with 0.4% ascorbate since weaning (i.e., that never experienced an ascorbate deficiency).

A comparable examination of the PCA for the proteomics data indicated that when we compared (1) the *Gulo*^*−/−*^ females treated with no ascorbate in drinking water for four weeks (FGL00: closed red circles), (2) the *Gulo*^*−/−*^ females treated with 0.4% ascorbate since weaning (FGL40: closed red squares), and (3) the *Gulo*^*−/−*^ females that experienced a four-week ascorbate depletion followed by a four-week 0.4% ascorbate treatment (FGLR40: open red squares), we observed an overlap between the proteomic profiles of the FGLR40 and FGL40 groups (Fig. 2C). A similar result was observed for the *Gulo*^*−/−*^ males (MGL00: closed blue circles; MGLR40: open blue squares; and MGL40: closed blue squares). These observations indicate that the proteomic profiles of ascorbate-deficient *Gulo*^*−/−*^ mice was re-established by a four-week treatment of 0.4% ascorbate in drinking water and was comparable to *Gulo*^*−/−*^ mice that never experienced an ascorbate deficiency.

Finally, in addition to a sexual dimorphism, the PCA results showed that ascorbate affects both the liver transcriptomic and proteomic profiles of *Gulo*^*−/−*^ mice.

### Identification of transcripts that showed significant correlations with ascorbate levels in female and male cohorts

**Fig. 3:**
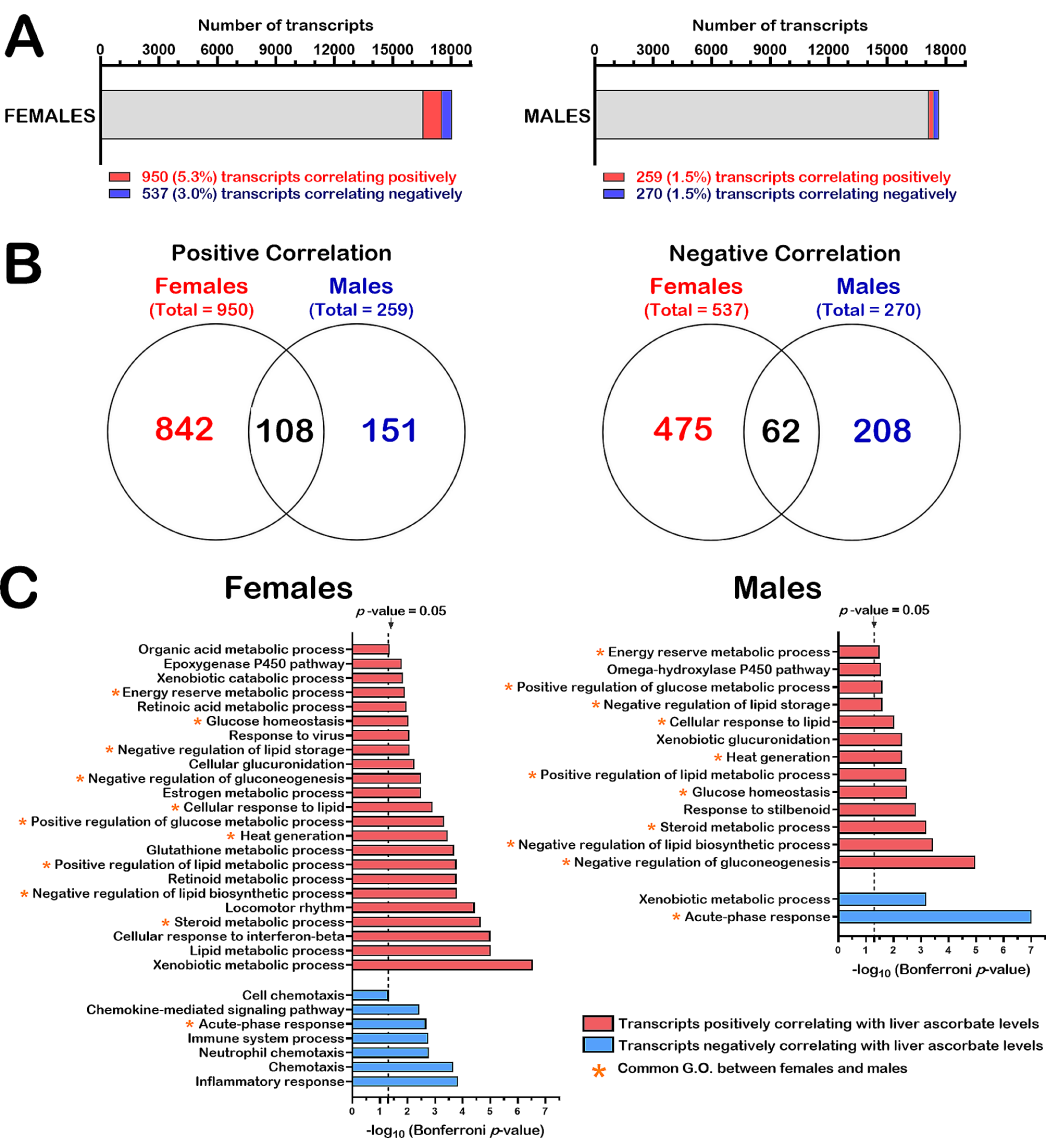
Liver transcripts correlating with hepatic ascorbate levels in females and males. (**A**) Graphs indicating the number and percentage of transcripts that correlated positively or negatively with hepatic ascorbate levels among the total number of identified and quantified transcripts in females (18,050 transcripts) and males (17,661 transcripts). (**B**) Venn diagrams showing the number of transcripts that correlated positively and negatively with liver ascorbate levels and the number of transcripts that were common among females and males. (**C**) Graphs showing biological processes with transcripts that significantly correlated positively or negatively with liver ascorbate levels in females and males. Asterisks indicate the processes that are common in both females and males

We next identified the genes for which transcripts levels were significantly correlating positively or negatively with liver ascorbate levels. Since we observed a sexual dimorphism, we independently analyzed the transcriptomics results obtained from females and males. Statistical associations between transcript levels and liver ascorbate levels were determined by Spearman correlation analyses with a specific set of stringent criteria. First, we only considered the transcripts with a TPM value different from zero in at least 70% of the samples for each gene. As such, if we independently analyzed the female samples, there was a total of 18,050 transcripts with TPM values different than 0 in > 70% of the female individuals (for *N* = 18 females). If we considered only the male samples, there was a total of 17,661 transcripts with TPM values different than 0 in > 70% of the male individuals (for *N* = 18 males) (Additional file 5: Table S3). Secondly, we also focused on transcripts that were different with at least a two-fold change between ascorbate-deficient *Gulo*^*−/−*^ mice (FGL00 or MGL00) and *Gulo*^*−/−*^ mice treated with 0.4% ascorbate in drinking water since weaning (FGL40 or MGL40). Finally, Spearman correlation was considered significant with a *p*-value < 0.05 for *N* = 18 females or males. Based on these criteria, 950 transcripts (corresponding to ∼ 5.3% of total quantified transcripts) and 537 (∼ 3.0%) transcripts in females correlated positively and inversely with hepatic ascorbate levels, respectively (Fig. 3A). In males, 259 transcripts (corresponding to ∼ 1.5% of total quantified transcripts) and 270 (∼ 1.5%) transcripts correlated positively and inversely with hepatic ascorbate levels, respectively (Fig. 3A). The complete lists of these transcripts are presented in the Additional file 10: Table S8 for females and males. These results indicated that more transcripts were affected by hepatic ascorbate in females than in males. Note that despite this observed sexual dimorphism, the Fig. 3B shows that 108 and 62 liver transcripts in both females and males correlated positively and inversely with hepatic ascorbate levels, respectively. The Additional file 11: Table S9 provides a list of such commonly altered transcripts.

We next determined which biological processes were enriched using the list of transcripts that correlated significantly with hepatic ascorbate in females and then in males (Fig. 3C, Bonferroni *p*-value < 0.05). Gene ontology analysis confirmed that more biological processes were affected by ascorbate levels in females (30 functional modules) than in males (15 modules). Biological processes common in both females and males included the energy reserve metabolic process, glucose homeostasis, negative regulation of lipid storage, negative regulation of gluconeogenesis, cellular response to lipid, positive regulation of glucose metabolic process, heat generation, positive regulation of lipid metabolic process, negative regulation of lipid biosynthetic process, and steroid metabolic process for the transcripts that correlated positively with liver ascorbate levels. The acute-phase response was the only common biochemical pathway that inversely correlated with hepatic ascorbate levels in both males and females (Fig. 3C). The Additional file 12: Table S10 provides a list of transcripts associated to these different biological processes.

### Identification of proteins for which LFQ intensities showed significant correlations with ascorbate levels in female and male cohorts

**Fig. 4:**
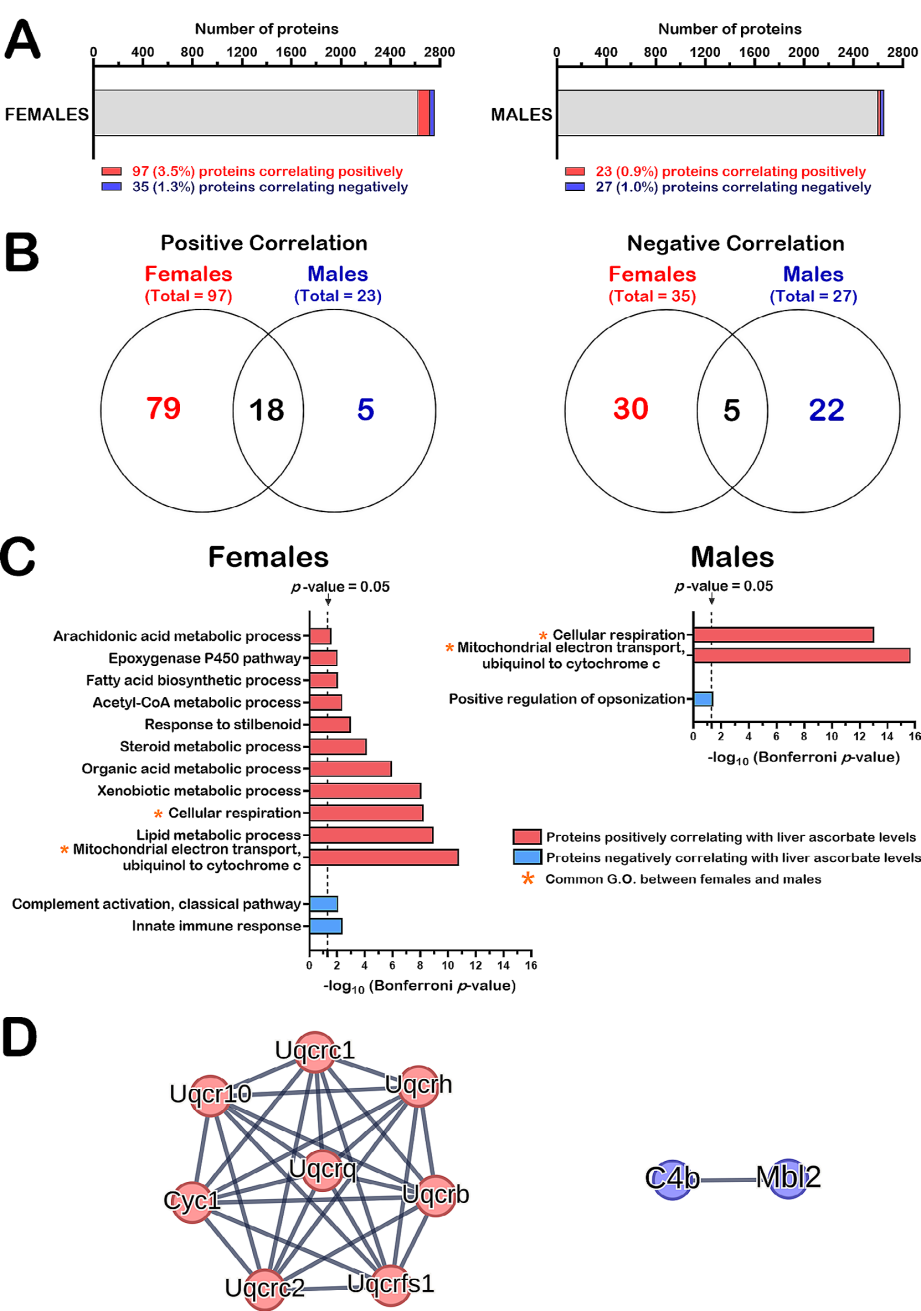
Liver proteins correlating with hepatic ascorbate levels in females and males. (**A**) Graphs indicating the number and percentage of proteins that correlated positively or negatively with hepatic ascorbate levels among the total number of identified and quantified proteins in females (2,900 proteins) and males (2,821 proteins). (**B**) Venn diagrams showing the number of proteins that correlated positively and negatively with liver ascorbate levels and that were common among females and males. (**C**) Graphs showing the biological processes of sets of proteins significantly correlating positively or negatively with liver ascorbate levels in females and males. (**D**) Protein-protein interaction networks of common proteins in females and males correlating positively (panel on the left, GO:0005750 mitochondrial complex III of the electron transport chain with a false discovery rate = 4.01 × 10^− 18^) or negatively with liver ascorbate levels using STRING (panel on the right, MMU-166,663 initial triggering of complement with a false discovery rate = 0.0194)

As for the transcriptomic data, females and males were separated into two different groups (of 18 animals each) for the Spearman correlation analyses. We thus searched in our lists of 3,414 identified proteins from all the liver samples, the number of proteins that were quantifiable in females and males separately. If only the female groups were considered, 2,900 proteins with at least two peptides were quantifiable. If only the male groups were considered, 2,821 proteins with at least two peptides were quantifiable (Additional file 13: Table S11). Statistical associations between LFQ intensities of the quantified proteins and the liver ascorbate levels were determined by Spearman correlation analyses with a specific set of stringent criteria. First, we only considered the proteins with at least 70% of valid values in the cohorts of females or males. Thus, a total of 2,758 and 2,648 proteins in females and males, respectively, passed this first criterion (Fig. 4A). Secondly, proteins with at least a two-fold change between the ascorbate-deficient mice and 0.4% ascorbate treated *Gulo*^*−/−*^ mice since weaning were specifically selected for the Spearman analysis. Finally, Spearman correlation was considered significant with a *p*-value < 0.05 for *N* = 18 females or males. Based on these criteria, 97 proteins (corresponding to ∼ 3.5% of proteins) and 35 (∼ 1.3%) proteins in females correlated positively and inversely with hepatic ascorbate levels, respectively (Fig. 4A). In males, 23 (corresponding to ∼ 0.9% of proteins) and 27 (∼ 1.0%) proteins correlated positively and inversely with hepatic ascorbate levels, respectively (Fig. 4A). The lists of these proteins are presented in the Additional file 14: Table S12 for females and males. These results indicated that more proteins were correlating with liver ascorbate in females than in males. Nevertheless, the Fig. 4B also reveals that eighteen and five liver proteins in both females and males correlated positively and inversely with hepatic ascorbate levels, respectively. These proteins are listed in Table 1.

**Table 1:** Proteins correlating with ascorbate levels in both females and males

Symbol	*rho* for females	*rho* for males
Fth1	0.76200	0.70108
Ftl1	0.73929	0.72690
Uqcrfs1	0.72211	0.72262
Slco1a1	0.71333	0.57202
Cyp2f2	0.71038	0.62087
Uqcrq	0.70934	0.69592
Cyc1	0.69421	0.74445
Uqcrc2	0.68905	0.76097
Nudt7	0.68766	0.49148
Fasn	0.68388	0.47521
Uqcrc1	0.66495	0.68388
Uqcr10	0.65462	0.76820
Ttc39c	0.64533	0.63430
Pklr	0.63326	0.61332
Mup3	0.62158	0.66288
Uqcrb	0.58028	0.72483
Cyp4a12a	0.56582	0.70212
Uqcrh	0.53278	0.63500
C4b	-0.49587	-0.66632
Hist1h1b	-0.55756	-0.49871
Stat3	-0.59607	-0.53795
Mbl2	-0.79608	-0.67011
Cdo1	-0.81612	-0.79608

We next determined which biological processes were enriched using the list of proteins that correlated significantly with hepatic ascorbate in females or males (Fig. 4C, Bonferroni *p*-value < 0.05). Gene ontology analysis confirmed that more biological processes were affected by ascorbate levels in females (13 functional modules) than in males (3 modules). Biological processes common in both females and males included cellular respiration and mitochondrial electron transport from ubiquinol to cytochrome c (Fig. 4C). The Additional file 15: Table S13 provides a list of proteins associated to these different biological processes.

To further highlight the functional links between the proteins that negatively and positively correlated with liver ascorbate levels in both females and males (Table 1), we used STRING (Search Tool for the Retrieval of Interacting Genes) [47]. Two of the five proteins that correlated inversely with liver ascorbate levels (C4b and Mbl2) were functionally linked to complement activation with a false discovery rate of 0.0194 (Fig. 4D, panel on the right). Eight of the 18 proteins that correlated positively with ascorbate levels in both females and males were functionally linked as they are part of the mitochondrial complex III of the electron transport chain and included Uqcrh, Uqcrb, Uqcrc1, Uqcrc2, Uqcr10, Uqcrq, Uqcrfs1, and Cyc1 (Fig. 4D, panel on the left). Relevant to the liver functions, STRING analysis also indicated that nine proteins correlating positively with hepatic ascorbate levels are associated with non-alcoholic fatty liver disease (NAFLD) with a false discovery rate of 5.81 × 10^− 13^. These proteins included the same subunits of the mitochondrial complex III of the electron transport chain mentioned above and the liver form of the enzyme pyruvate kinase (Pklr).

### Combined transcriptomic and proteomic analysis

**Fig. 5:**
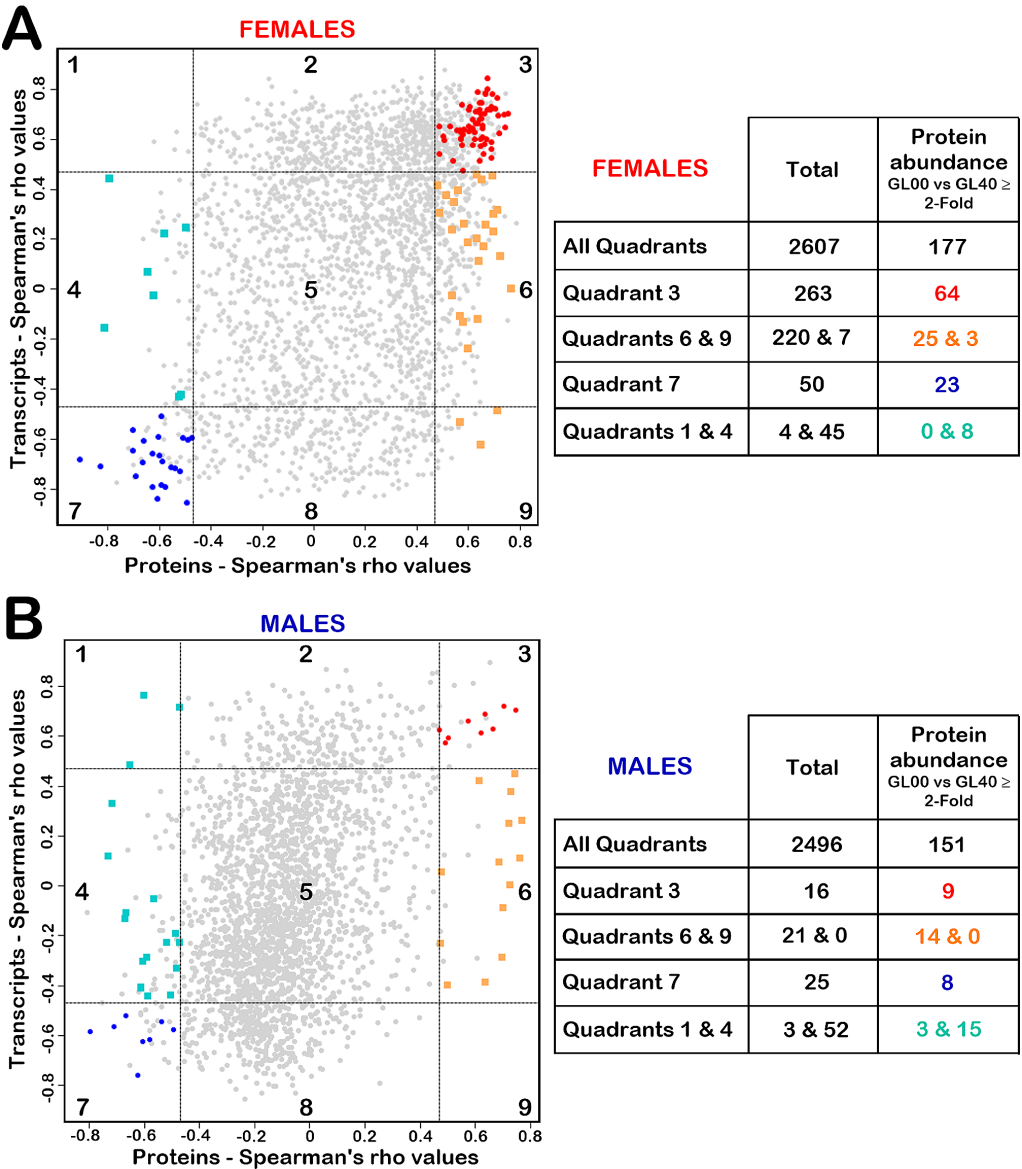
Nine-quadrant diagrams of transcriptome and proteome Spearman correlation analysis in females (**A**) or males (**B**). The scatter plots, on the left panels, are divided into nine quadrants with the x and y axes representing the Spearman *rho* values for the proteins and the transcripts, respectively, using the significance thresholds (dashed lines on the x and y axes at the *rho* values equal to 0.46876 or -0.46876). The quadrant numbers are included in each rectangle in black on the scatter plots. Genes that exhibited at least a two-fold abundance difference, at their protein level, between GL00 and GL40, were represented by red circles in quadrant 3, blue circles in quadrant 7, turquoise squares in quadrants 1 and 4, and orange squares in quadrants 6 and 9. The number of proteins in each quadrant are shown in the tables on the right of the scatter plots for the females (**A**) and males (**B**)

To determine whether ascorbate modulated protein abundance at the transcriptional or post-transcriptional level in the whole liver of our mouse cohorts, nine-quadrant scatter plots were created with the Spearman’s *rho* values of the proteins and transcripts (Fig. 5). We first generated a list of identified proteins that had a corresponding transcript with quantifiable information in females or males. Note that only transcripts and proteins with more than 70% valid values were considered. A total of 2,607 and 2,496 proteins had a corresponding transcript in females and males, respectively (Additional file 16: Table S14). The scatter plots were divided into nine quadrants with the x and y axes representing the Spearman correlation *rho* values for the proteins and the transcripts, respectively. The Spearman *rho* values with *p*-values < 0.05 were set to screen out the transcripts and proteins that did not significantly correlated with hepatic ascorbate levels. The dashed lines on the x and y axes indicated the significance thresholds at the protein and transcript levels, respectively. The genes in quadrant 5 for both females (Fig. 5A) and males (Fig. 5B) were not significantly correlating with hepatic ascorbate at both the transcript and protein levels. The quadrants 2 and 8 showed genes that significantly correlated with hepatic ascorbate at their transcript level while they did not significantly correlate with hepatic ascorbate at their protein level. Ascorbate may modulate the genes included in quadrant 2 and 8 at the transcriptional level or may impact on the stability of their transcripts, but the protein abundance of each of these genes was not reflected by such modulations in the whole liver lysates. Genes in quadrant 3 showed a significant positive correlation with ascorbate at both transcript and protein levels while genes in quadrant 7 were significantly negatively correlating with ascorbate at both transcript and protein levels. Thus, synchronous changes in transcription and protein levels for genes located in quadrants 3 and 7 in females (Fig. 5A) and males (Fig. 5B) were observed and may represent transcriptional regulation of protein levels by ascorbate. The quadrants 4 and 6 showed genes that significantly correlated with hepatic ascorbate at their protein level while they did not significantly correlate with hepatic ascorbate at their transcript level. The quadrants 1 and 9 showed genes that significantly correlated with hepatic ascorbate at both transcript and protein level but in an opposite direction. For example, proteins in quadrant 1 correlated negatively with ascorbate levels while their corresponding transcripts correlated positively. Thus, the proteins located in quadrants 1, 4, 6, and 9 likely represented proteins which abundance was potentially modulated by ascorbate at a post-transcriptional level in females (Fig. 5A) and males (Fig. 5B).

To determine which biological processes were enriched using the list of proteins that showed transcriptional or post-transcriptional modulations by ascorbate levels, we focused on the proteins that exhibited at least a two-fold abundance difference between ascorbate-deficient *Gulo*^*−/−*^ mice (FGL00 and MGL00) and *Gulo*^*−/−*^ mice treated with 0.4% ascorbate in drinking water since weaning (FGL40 and MGL40). Such proteins were represented by red circles in quadrant 3, blue circles in quadrant 7, turquoise squares in quadrants 1 and 4, orange squares in quadrants 6 and 9. The number of proteins in each quadrant are shown in the tables on the right of the scatter plots for the females (Fig. 5A) and males (Fig. 5B). The lists of all the proteins in the nine-quadrant plots are presented in the Additional file 16: Table S14 for females and males. As indicated in Table 2, seven biological processes were positively regulated by ascorbate in females. These biological processes were composed of genes for which both their transcripts and proteins significantly correlated positively with ascorbate levels (quadrant 3). In addition, two biological processes were inversely regulated by ascorbate in females. Such biological processes were composed of genes for which both their transcripts and proteins significantly correlated negatively with ascorbate levels (quadrant 7). In contrast, no biological process was significantly enriched in males as there were much fewer genes that had both their transcripts and proteins correlating with whole liver ascorbate levels compared to females (quadrants 3 and 7 in Fig. 5A and B).

**Table 2:** Biological processes from proteins modulated by hepatic ascorbate levels during the transcriptional process or post-transcriptionally

**Biological processes**	Bonferroni *p*-value	Proteins
**Females**		
**Proteins at least modulated at the transcriptional level by ascorbate**		
**Positive regulation (quadrant 3)**		
Lipid metabolic process	4.18E-08	Abcd2, Acacb, Aacs, Acaca, Cyp17a1, Cyp39a1, Acly, Fads2, Ces2c, Sult1d1, Rdh11, Fasn, Aldh1a1, Sult2a8, Cept1, Ugt1a9, Mgll
Xenobiotic metabolic process	1.61E-07	Cyp2d10, Cyp2a4, Cyp2a12, Cyp2b10, Gsta2, Sult2a8, Fmo1, Cyp2f2, Cyp2d26
Organic acid metabolic process	2.56E-06	Cyp2d10, Cyp2a4, Cyp2a12, Cyp2b10, Fmo1, Cyp2f2, Cyp2d26
Fatty acid biosynthetic process	7.87E-04	Acly, Fads2, Fasn, Acacb, Acaca, Mgll
Acetyl-CoA metabolic process	8.66E-04	Acly, Fasn, Acacb, Acaca
Steroid metabolic process	9.71E-04	Cyp39a1, Ugt2b1, Cyp2b10, Akr1c14, Cyp3a44, Sult2a8, Cyp17a1
Response to stilbenoid	0.0108	Cyp2a4, Slco1a1, Gsta2, Mup3
**Negative regulation (quadrant 7)**		
Inflammatory response	0.0214	C4b, Stat3, Pld4, Chil3, S100a9, S100a8
Innate immune response	0.0314	C4b, Ighg2b, Ighg2c, Igkc, Pld4, S100a9, S100a8
**Proteins modulated at the post-transcriptional level by ascorbate**		
**Positive regulation (quadrants 6 & 9)**		
Mitochondrial electron transport ubiquinol to cytochrome c	9.56E-16	Uqcrb, Uqcrq, Uqcrc1, Uqcrfs1, Uqcr10, Uqcrc2, Cyc1, Uqcrh
Cellular respiration	3.65E-13	Uqcrb, Uqcrq, Uqcrc1, Uqcrfs1, Uqcr10, Uqcrc2, Cyc1, Uqcrh
**Negative regulation (quadrants 1 & 4)**		
No significant term		
**Males**		
**Proteins at least modulated at the transcriptional level by ascorbate**		
**Positive regulation (quadrant 3)**		
No significant term	-	-
**Negative regulation (quadrant 7)**		
No significant term	-	-
**Proteins modulated at the post-transcriptional level by ascorbate**		
**Positive regulation (quadrants 6 & 9)**		
Mitochondrial electron transport ubiquinol to cytochrome c	2.05E-18	Uqcrb, Uqcrq, Uqcrc1, Uqcrfs1, Uqcr10, Uqcrc2, Cyc1, Uqcrh
Cellular respiration	7.81E-16	Uqcrb, Uqcrq, Uqcrc1, Uqcrfs1, Uqcr10, Uqcrc2, Cyc1, Uqcrh
Metabolic process	0.0248	Pfkfb1, Casp7, Pklr, Fasn
**Negative regulation (quadrants 1 & 4)**		
Positive regulation of opsonization	0.0230	C3, C4b, Mbl2

Two-way ANOVA were performed on the whole data set to determine the influence of sex and ascorbate treatments on proteins abundances. Overall, 39 proteins were part of the biological processes identified from quadrants 3 and 7 in females (Table 2). Eight of these proteins were quantifiable only in females and thus a two-way ANOVA could not be performed. Twenty-four of these proteins were influenced by sex (with a *p*-value < 0.05). Twenty-eight proteins were influenced by ascorbate treatments. Nine proteins showed a significant interaction between sex and ascorbate treatments (Additional file 17: Table S15). Thus, the two-way ANOVA corroborated the identification of ascorbate dependent biological processes that were affected only in the females.

We next determined the biological processes associated with the sets of proteins that correlated with hepatic ascorbate levels unlike their corresponding transcripts in both females and males. Two biological pathways were enriched for proteins that correlated positively with ascorbate levels in the females (quadrants 6 and 9 in Table 2). Three biological processes were enriched for proteins that correlated positively with ascorbate levels in the males (Table 2). Two of these pathways were common between males and females and included mitochondrial electron transport from ubiquinol to cytochrome c and cellular respiration. Eight proteins were part of these biochemical pathways in both males and females and included Uqcrh, Uqcrb, Uqcrc1, Uqcrc2, Uqcr10, Uqcrq, Uqcrfs1, and Cyc1. Two-way ANOVA indicated that all these eight proteins were influenced by ascorbate treatments in both males and females (Additional file 17: Table S15). Uqcrq was the only protein influenced by sex. However, there was no interaction between sex and ascorbate treatment. Importantly, these eight proteins were the same uncovered by STRING analysis in Fig. 4D and are part of the mitochondrial complex III of the electron transport chain. Thus, the combined transcriptomic and proteomic analyses indicated that the protein levels of several subunits of the mitochondrial complex III decreased in ascorbate-deficient *Gulo*^*−/−*^ mice, despite no observable change of their corresponding transcripts. Finally, two-way ANOVA validated the influence of ascorbate on these eight proteins regardless of the sex of the mice.

No biological pathway was significantly enriched (with a Bonferroni *p*-value < 0.05) for proteins that correlated inversely with ascorbate levels in the females or males (quadrants 1 and 4). Mbl2 was the only protein common between males and females in quadrants 1 and 4 suggesting a post-transcriptional regulation of this protein by ascorbate.

### Quantitative RT-PCR analyses and immunoblotting of various mitochondrial complex III subunits

**Fig. 6:**
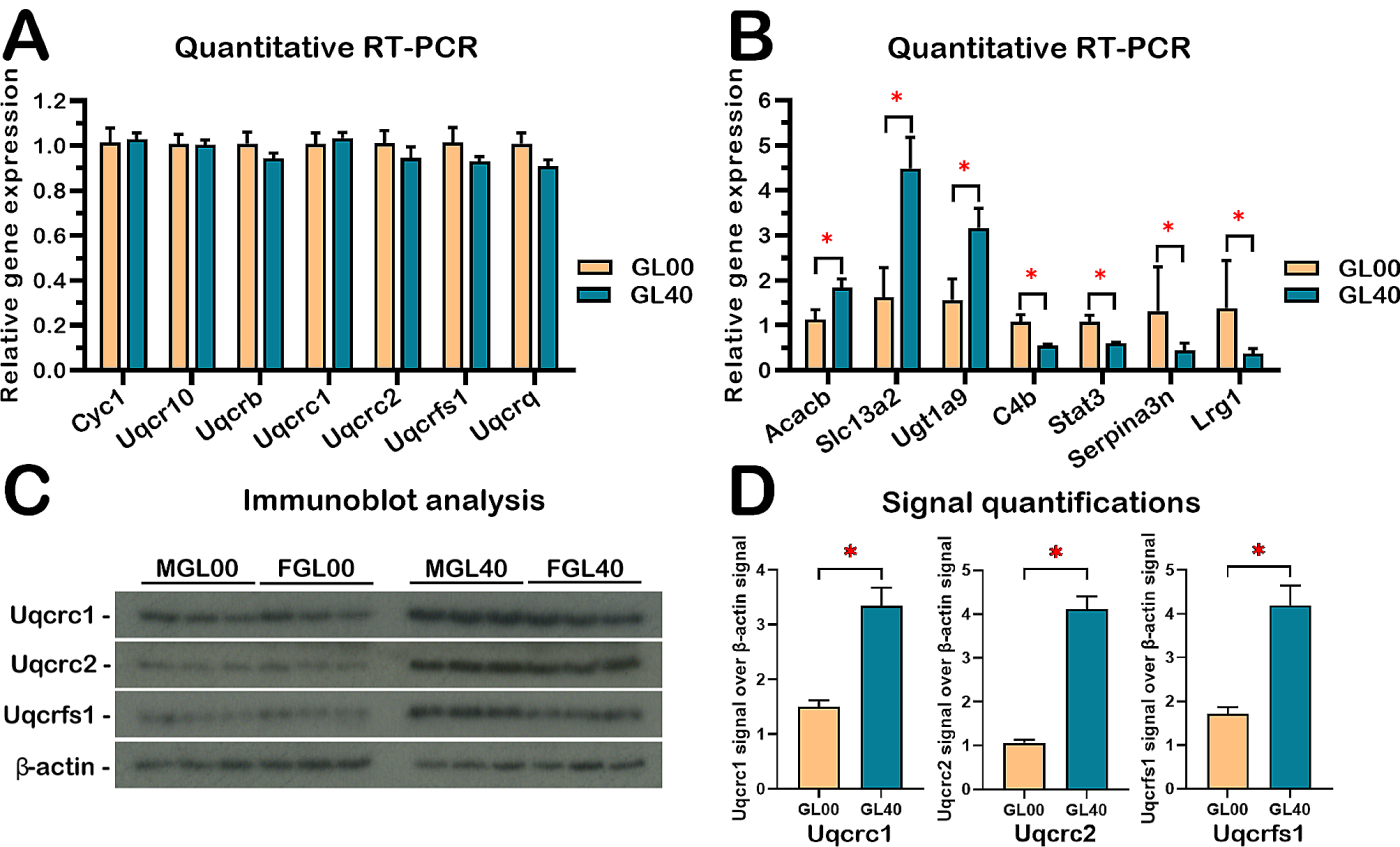
Quantitative RT-PCR analyses and immunoblotting in ascorbate-deficient *Gulo-/-* mice livers compared to *Gulo*^*−/−*^ mice treated with 0.4% ascorbate in drinking water since weaning. (**A**) Graph showing the expression of seven mRNAs coding for mitochondrial complex III complex proteins by quantitative RT-PCR in the liver of both males and females. GL00 = ascorbate-deficient *Gulo*^*−/−*^ mice; GL40 = *Gulo*^*−/−*^ mice treated with 0.4% ascorbate since weaning (*N* = 4 for each RT-PCR experiment). (**B**) Graphs of quantitative RT-PCR analyses of the indicated gene transcripts correlating positively or negatively with liver ascorbate levels in *Gulo*^*−/−*^ mice treated with 0.4% ascorbate in drinking water since weaning compared to ascorbate-deficient *Gulo*^*−/−*^ mice (*N* = 4 for each RT-PCR experiment) (*p-value < 0.05; Welch’s *t*-test). (**C**) Analyses of three different proteins from the mitochondrial complex III of the electron transfer chain complexes by western blots on whole liver lysates for *Gulo*^*−/−*^ males and females (*N* = 3 females (F) and 3 males (M) for each cohort). (**D**) Signal quantification of Uqcrc1, Uqcrc2, and Uqcrfs1 proteins over ß-actin signal from the immunoblot analysis in **C** (*N* = 6 animals in total per groups)

Since the combined transcriptomic and proteomic analyses indicated that the protein levels of several subunits of the mitochondrial complex III decreased in ascorbate-deficient *Gulo*^*−/−*^ mice despite no observable change of their corresponding transcripts, we first performed quantitative RT-PCR on total liver RNA to confirm that indeed no change was observed at the transcriptional level. Atp5o and Ubc were used as control housekeeping genes (no difference of expression between females or males GL00 and GL40 groups). The Additional file 18: Table S16 provides the primer sequences for the different genes analyzed. As indicated in Fig. 6A, there was no difference at the RNA level for Uqcrb, Uqcrc1, Uqcrc2, Uqcr10, Uqcrq, Uqcrfs1, and Cyc1 between ascorbate-deficient *Gulo*^*−/−*^ mice (GL00) and *Gulo*^*−/−*^ mice treated with 0.4% ascorbate (GL40) with the combined male and female samples. The RT-PCR results confirmed the transcriptomic data for these mice regarding these seven genes. Additional quantitative RT-PCR was done for seven genes on total RNA from ascorbate-deficient *Gulo*^*−/−*^ mice (GL00) and *Gulo*^*−/−*^ mice treated with 0.4% ascorbate in drinking water since weaning (GL40) to ensure that we were able to detect transcriptional alterations between both cohorts of mice. We selected seven transcripts that exhibited no significant abundance difference between males and females (no sexual dimorphism) and showed similar increase or decrease between GL00 and GL40 groups in both males and females. In agreement with the RNA-seq data, the quantitative RT-PCR analyses indicated that the Acacb, Slc13a2, and Ugt1a9 transcript levels were significantly down regulated in both the ascorbate-deficient *Gulo*^*−/−*^ females and males (GL00) compared to the 0.4% ascorbate treated *Gulo*^*−/−*^ females and males (GL40) (Fig. 6B). The C4b, Lrg1, Serpina3n, and Stat3 transcript levels were significantly up regulated in both the ascorbate-deficient *Gulo*^*−/−*^ females and males (GL00) compared to the 0.4% ascorbate treated *Gulo*^*−/−*^ females and males (GL40) (Fig. 6B).

Western blots with appropriate antibodies against Uqcrc1, Uqcrc2, and Uqcrfs1 showed significant level differences between GL00 and GL40 in both females and males in the whole liver lysates as observed with the LFQ data (Fig. 6C and D). All these results confirmed that the difference in the protein levels of several subunits of the mitochondrial complex III was not due to a difference in their mRNA levels suggesting a post-transcriptional regulation by ascorbate in the liver of both females and males.

### Polysome profiling followed by targeted quantitative RT-PCR analyses of mRNAs coding for mitochondrial complex III subunits and the mannose binding lectin 2 (Mbl2)

**Fig. 7:**
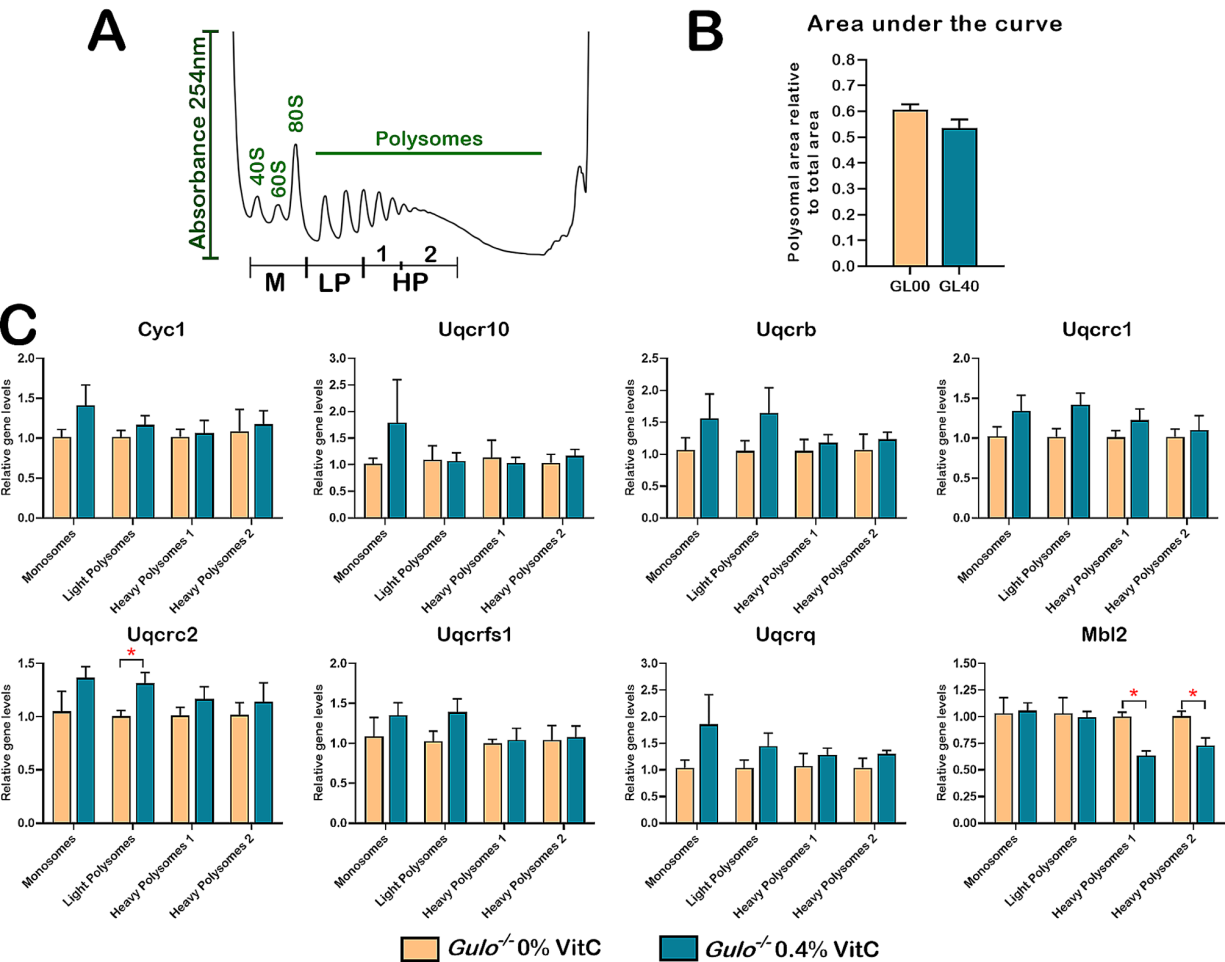
Polysome profiling and targeted quantitative RT-PCR analyses on mRNAs from the different polysomal fractions. (**A**) Example of a polysomal profile obtained from a *Gulo*^*−/−*^ male treated with 0.4% ascorbate (M = monosome, LP = light polysome, HP = heavy polysome). (**B**) Quantification of area-under-curve for polysomal area normalized to total area. *N* = 4–5 males per groups. (**C**) Graphs showing the relative abundance of seven mRNAs coding for mitochondrial complex III complex proteins and mRNA coding for Mbl2 by quantitative RT-PCR in the different polysomal fractions extracted from male liver tissues. GL00 = ascorbate-deficient *Gulo*^*−/−*^ mice; GL40 = *Gulo*^*−/−*^ mice treated with 0.4% ascorbate since weaning (*p-value < 0.05; Welch’s *t*-test)

To investigate the discrepancy between the mRNA and the protein levels of different mitochondrial complex III subunits, we isolated mRNAs using the polysome profiling method. The association of mRNAs with polysomes provides an insight into protein production by assessing the mRNAs specifically involved in active translation. Using liver tissues from ascorbate-deficient *Gulo*^*−/−*^ males and 0.4% ascorbate-treated *Gulo*^*−/−*^ males, we were able to obtain polysome profiles on sucrose gradient preparations for each animal. The Fig. 7A presents an example of the polysome profiles obtained for one *Gulo*^*−/−*^ male treated with 0.4% ascorbate. The polysome profiles of all the tested males are depicted on the Additional file 19: Figure S3. We subdivided polysome profile into four different fractions: monosomes, light polysomes, first and second heavy polysomes (Fig. 7A). Heavy polysomes are generally considered the most actively translating ribosomes. Overall, the polysome profiles of ascorbate-deficient *Gulo*^*−/−*^ males (MGL00) were similar to *Gulo*^*−/−*^ males treated with 0.4% ascorbate (MGL40). The ratio of polysomal area over total area indicated that there was no significant difference in general translation between the two groups (Fig. 7B).

We next performed quantitative RT-PCR on some genes that showed a significant difference in their protein abundances but not in their total mRNA levels in male livers. As indicated in Fig. 7C, the abundance of mRNAs encoding Cyc1, Uqcrc10, Uqcrb, Uqcrc1, Uqcrc2, Uqcrfs1, and Uqcrq proteins associating with the heavy polysomes fractions was not significantly different between MGL00 and MGL40 animals. There is thus no evidence for an enrichment of mRNAs coding for mitochondrial complex III proteins in fractions corresponding to actively translating heavy polysomes in MGL40 compared to MGL00 animals. In contrast, the level of mRNA coding for Mbl2 detected in heavy polysomes fractions of MGL40 mice was significantly decreased compared to MGL00 mice (Fig. 7C), even though the total mRNA levels of Mbl2 were the same between GL00 and GL40 groups of mice based on quantitative RT-PCR analysis (Additional file 20: Figure S4A). This result is concordant with the lower abundance of Mbl2 proteins detected by mass spectrometry in the liver of MGL40 compared to MGL00 mice (Additional file 9: Table S7 and Additional file 20: Figure S4B).

## Discussion

In the present study, we established a combined transcriptomic and proteomic profiling strategy on the whole liver of *Gulo*^*−/−*^ mice to highlight the specific molecular changes that took place during ascorbate deficiency at the transcriptional and post-transcriptional levels. The main strength of our study was not only to extract mRNAs and proteins from the same animals to minimize sampling bias between transcriptomic and proteomic data, but also to quantify the hepatic ascorbate levels from the same mice enabling direct Spearman correlation computation. Both the transcriptomic and proteomic data unquestionably depicted a separation of the overall hepatic transcriptome and proteome profiles between females and males (Fig. 2A and C). Additional gene ontology analyses revealed that six biological processes were affected at both the transcriptome and proteome levels even though our criteria were highly stringent in both omics analyses (i.e., at least a two-fold difference between females and males with an adjusted *p*-value < 0.05 for multiple comparisons). These biological processes included the xenobiotic metabolic process, lipid metabolic process, steroid metabolic process, epoxygenase P450 pathway, organic acid metabolic process, and response to stilbenoid. Noticeably, the observed sexual dimorphism with regards to gene expression is in accordance with other published results [48–53]. In the last two decades, reports have illustrated the profound effects of sex on the expression of genes particularly involved in the epoxygenase P450 pathway, the xenobiotic, organic acid, and steroid metabolic processes [48, 54–57], as well as liver disease susceptibility [56, 58, 59]. The sexual dimorphism observed in our PCAs also agrees with previous studies on *Gulo*^*−/−*^ mice treated with different concentrations of ascorbate [26, 31]. The precise reason for the differences between female and male transcriptome and proteome profiles is unclear but sexual hormone dimorphism is likely to play a major role in such distinctions [56, 58–60]. Accordingly, experiments on rats have revealed that micromolar concentrations of ascorbate increased the binding ability and the enzymatic activities of type II estrogen receptor [61]. In addition, in postmenopausal women on hormone replacement therapy, an ascorbic acid treatment of one month (1000 mg daily) successfully lead to the rise of estradiol levels, especially for women with an initial ascorbic acid plasma concentration inferior to 70 µmol/L [62]. Collectively, these studies suggested that vitamin C participates to the modulation of steroid hormone synthesis and to their downstream effects leading to sexual dimorphism. The results of the present study concur with these reports.

In addition to the hepatic sexual dimorphism, our PCAs allowed us to visualize how the general transcriptomes and the proteomes of our different mouse groups differed from one another depending on ascorbate treatments. Intriguingly, we observed that the efficiency of the four-week ascorbate rescue experiment on vitamin C deficient *Gulo*^*−/−*^ mice was different between the proteomic profiles (complete rescue) and the transcriptomic profiles (partial rescue). This difference may be due to the lower number of quantifiable proteins. Indeed, a major limitation of proteomic investigations remains the complexity of biological samples like a whole liver and as such various low abundance proteins can be much harder to detect using the current mass spectrometry technology [63, 64]. Although outside the scope of the present study, multi subcellular fractionation experiments on liver samples (including the identification of subcellular localization of proteins after a stress [65]) may help with the identification and quantification of low abundance proteins [66].

Importantly, our combined omics investigations and Spearman correlation analyses revealed that ascorbate modulates the abundance of various proteins at either the transcriptional or post-transcriptional levels, in vivo. Firstly, our results revealed that gene expression of many enzymes involved in fatty acid biosynthesis as well as xenobiotic, organic acid, acetyl-CoA, and steroid metabolism was primarily regulated at the transcriptional level. Importantly, the ascorbate deficiency in *Gulo*^*−/−*^ mice was thus associated with a decrease in these biological processes and coincided with the body weight loss and the decreased phospholipids and hexose levels previously observed in the serum of these mice [24, 25, 67]. Secondly, combining transcriptomic and proteomic Spearman correlation data revealed several proteins that correlated positively or inversely with hepatic ascorbate levels in females or males, whereas their corresponding identified transcripts did not correlate according to our stringent criteria (Fig. 5). We surmise that for such genes, the protein abundance regulation by ascorbate may occur post-transcriptionally. Thirdly, our polysomal profiling analysis indicated that the overall polysomal profiles did not significantly differ between ascorbate-deficient *Gulo*^*−/−*^ mice and *Gulo*^*−/−*^ mice treated with 0.4% ascorbate in drinking water since weaning (Fig. 7). Thus, we infer that ascorbate deficiency did not affect the global translation in the hepatic tissue of *Gulo*^*−/−*^ mice but may influence the translation of specific targeted liver mRNAs.

We do not know whether ascorbate directly or indirectly affects transcription and/or translation from the present study. The mechanism by which ascorbate modulates the expression of each individual gene may differ and should therefore be thoroughly examined. Intriguingly, we noticed that the expression of hydrolases or dioxygenases enzymes and several histone or DNA demethylases known to rely on ascorbate as a co-factor for their activity did not significantly change upon ascorbate deficiency among our groups of mice. Nevertheless, an ascorbate deficiency in *Gulo*^*−/−*^ mice may only affect the catalytic activity of these enzymes and not their proteins levels as they were not part of the list of proteins which abundance was altered by hepatic ascorbate levels. Thus, thorough epigenomics analysis in the liver of our mouse cohort will be required to determine which genes are affected in ascorbate deficient *Gulo*^*−/−*^ mice through the activity of histone or DNA demethylases. The analysis of transcription factors known to be modulated by their redox status will also be required as ascorbate could indirectly regulate such factors through its antioxidative properties [16].

To our knowledge, the present study indicates for the first time that ascorbate could regulate the abundance of specific proteins at a post-transcriptional level in vivo. Eighteen proteins correlated positively with ascorbate levels unlike their transcripts and included eight mitochondrial complex III subunits (Fig. 5C) in both females and males. In addition, five proteins (Mbl2, Cdo1, Stat3, Hist1h1b and C4b) correlated inversely with liver ascorbate levels unlike their transcripts in both females and males. Interestingly, Mbl2 is one example of gene that we successfully detected as being specifically regulated post-transcriptionally by ascorbate via a modulation of the association of the mRNA coding for Mbl2 with actively translating polysomes (Fig. 7). This result explains the discrepancy observed between our transcriptome and proteome results for Mbl2. Importantly, Mbl2 is one of the proteins related to the innate immune response and the complement activation that have been uncovered in both females and males as a biological process inversely correlated with liver ascorbate concentrations (Fig. 4B and D). Interestingly, a targeted mass spectrometry analysis has reported that the abundance of several factors of the innate immune response are inversely associated with plasma ascorbate levels in humans [68]. Thus, an increase immune activity in response to an ascorbate deficiency is observable in both humans and our *Gulo*^*−/−*^ mouse model.

In contrast to the conclusion drawn for Mbl2 gene regulation by ascorbate, quantitative RT-PCR analysis of mRNA isolated from ribosomal fractions across the polysome profiles indicated that all the mRNAs coding for the mitochondrial complex III subunits tested were equally abundant in each fraction obtained from either ascorbate-deficient *Gulo*^*−/−*^ mice or *Gulo*^*−/−*^ mice treated with 0.4% ascorbate since weaning (Fig. 7C). However, western blot analyses on the liver tissues of both female and male *Gulo*^*−/−*^ mice treated with sub-optimal concentrations of ascorbate revealed the lower protein levels of several mitochondrial complex III subunits compared to *Gulo*^*−/−*^ mice treated with optimal concentrations of vitamin C, even though the level of their corresponding transcripts was not different between the two groups (Fig. 6). We infer that the modulation of the abundance of the different mitochondrial complex III subunits by ascorbate is the consequence of a post-transcriptional regulation.

Interestingly, even though ascorbate-deficient *Gulo*^*−/−*^ mice do not exhibit hepatic steatosis with normal chow [69], STRING analysis uncovered that several mitochondrial complex III subunits correlating positively with hepatic ascorbate levels were associated to non-alcoholic fatty liver disease (NAFLD). Of relevance, recent studies unveiled that dysfunctional mitochondria participate in the aggravation of NAFLD [28]. It has been reported that the progression of NAFLD to non-alcoholic steatohepatitis is accompanied by a decrease mitochondrial respiratory chain including a 70% decrease of complex III activity in human patients [70]. Importantly, the lower protein levels of mitochondrial complex III in ascorbate-deficient *Gulo*^−/−^ mice was also associated with a decrease in the activity of this complex in the liver [31]. These results suggest that an ascorbate deficiency leads to a mitochondrial dysfunction that can exacerbate the progression of NAFLD especially in subjects with an unbalanced diet. Accordingly, a diet poor in legumes and fruits (and thus implicating a low vitamin C intake) and high in fatty and sugary processed foods has been associated with increased risk of NAFLD [71–75]. Furthermore, a study revealed that a twelve-week oral ascorbate supplementation succeeded in elevating plasma vitamin C concentrations and improving liver health in patients with NAFLD [76]. To date, no study in human has collected the information on both the plasma vitamin C concentration and the protein levels or activity of the mitochondrial complex III in patients with NAFLD in comparison to healthy individuals. Such investigations could reveal that ascorbate deficiency is a major risk factor for mitochondrial dysfunction that could exacerbate to liver pathogenesis.

## Conclusions

In the current investigation, we used *Gulo*^*−/−*^ female and male mice, which cannot synthesize their own ascorbate, to determine the impact of this vitamin on both the transcriptomics and proteomics profiles in the whole liver at the age of four months. Using Spearman correlation analyses, we unveiled transcripts encoding a wide array of biological processes involved in glucose and lipid metabolisms as well as in the acute-phase immune response correlating with hepatic ascorbate levels. Moreover, integration of the proteomics data showed that ascorbate modulates the abundance of various enzymes involved in lipid, xenobiotic, organic acid, acetyl-CoA, and steroid metabolism mainly at the transcriptional level in females while the abundances of the mitochondrial complex III subunits were regulated post-transcriptionally by ascorbate in the liver of both females and males. Of relevance, the loss of mitochondrial complex III activity has been reported in human NAFLD condition [70]. We thus infer that an hypovitaminosis C would exacerbate hepatic mitochondrial dysfunction in NAFLD subjects.

### Electronic supplementary material

Below is the link to the electronic supplementary material.

Additional file 1: Table S1

Additional file 2: Figure S1

Additional file 3: Table S2

Additional file 4: Figure S2

Additional file 5: Table S3

Additional file 6: Table S4

Additional file 7: Table S5

Additional file 8: Table S6

Additional file 9: Table S7

Additional file 10: Table S8

Additional file 11: Table S9

Additional file 12: Table S10

Additional file 13: Table S11

Additional file 14: Table S12

Additional file 15: Table S13

Additional file 16: Table S14

Additional file 17: Table S15

Additional file 18: Table S16

Additional file 19: Figure S3

Additional file 20: Figure S4

## Data Availability

The transcriptomic data have been deposited in NCBI’s Gene Expression Omnibus and are accessible through GEO: GSE233598. All mass spectrometry data (raw files and MaxQuant search result files) are publicly available on ProteomeXchange repository (www.proteomexchange.org) with the identifier PXD042513.

## Abbreviations

Acacb: Acetyl-Coenzyme A carboxylase beta
Adra2a: Adrenergic receptor, alpha 2a
AGC: Automated gain control
Atp5o: ATP synthase, H + transporting, mitochondrial F1 complex, O subunit
C4b: Complement component 4B
Cdo1: Cysteine dioxygenase 1
Cyc1: Cytochrome c-1
DAVID: Database for Annotation, Visualization and Integration Discovery
FDR: False discovery rate
GL01: *Gulo*^*−/−*^ females or males treated with 0.01% ascorbate for 4 months
GL05: *Gulo*^*−/−*^ females or males treated with 0.05% ascorbate for 4 months
GL40: *Gulo*^*−/−*^ females or males treated with 0.4% ascorbate for 4 months
GLR40: *Gulo*^*−/−*^ females or males treated with 0% ascorbate for 1 month followed by 1 month of 0.4% ascorbate treatment
GO: Gene ontology
GULO: Gulonolactone oxidase
Hist1h1b: H1.5 linker histone, cluster member
HP: Heavy polysome
LFQ: Label free quantification
LP: Light polysome
Lrg1: Leucine-rich alpha-2-glycoprotein 1
M: Monosome
Mbl2: Mannose-binding lectin 2
PCA: Principal component analysis
Pklr: Pyruvate kinase liver and red blood cell
Slc13a2: Solute carrier family 13 (sodium-dependent dicarboxylate transporter), member 2
Stat3: Signal transducer and activator of transcription 3
STRING: Search tool for the retrieval of interacting genes
TPM: Transcripts per kilobase million
Ubc: Ubiquitin C
Uqcr10: Ubiquinol-cytochrome c reductase, complex III subunit X
Ugt1a9: UDP glucuronosyltransferase 1 family, polypeptide A9
Uqcrb: Ubiquinol-cytochrome c reductase binding protein
Uqcrc1: Ubiquinol-cytochrome C reductase core protein I
Uqcrc2: Ubiquinol-cytochrome C reductase core protein II
Uqcrfs1: Ubiquinol-cytochrome c reductase, Rieske iron-sulfur polypeptide 1
Uqcrh: Ubiquinol-cytochrome c reductase hinge protein
Uqcrq: Ubiquinol-cytochrome c reductase complex III subunit VII
VitC: vitamin C
WT00: WT females or males with no ascorbate for 4 months
GL00: *Gulo*^*−/−*^ females or males with no ascorbate for 1 month
