# Supplementary figures and images for: Combined transcriptomics and proteomics unveil the impact of vitamin C in modulating specific protein abundance in the mouse liver
Source: Biol Res. 2024 May 12;57:26. doi: 10.1186/s40659-024-00509-x (PMC11088995; doi:10.1186/s40659-024-00509-x)

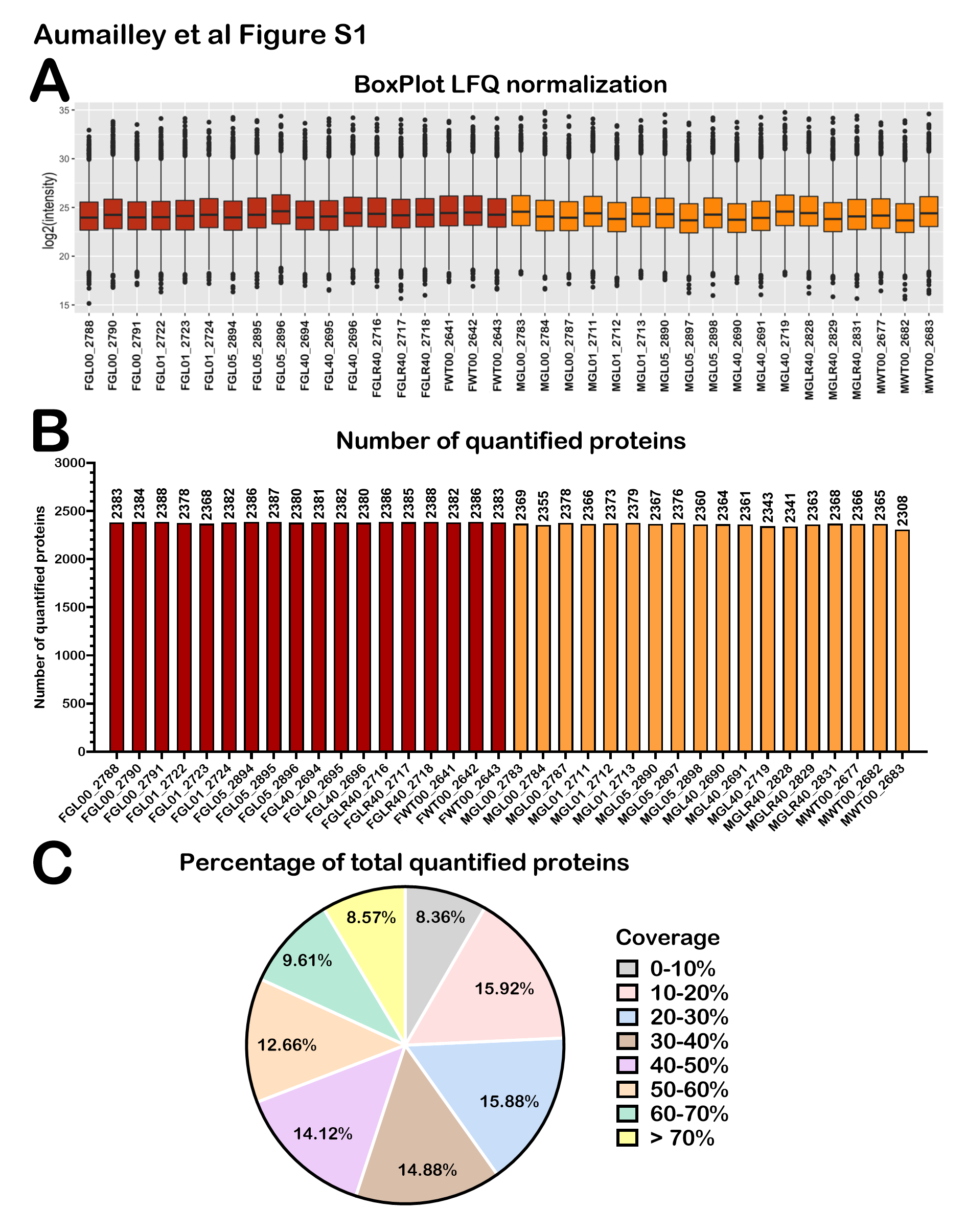

Supplement: Supplementary file 2 — Additional file 2: Figure S1 [file 40659_2024_509_MOESM2_ESM.png]

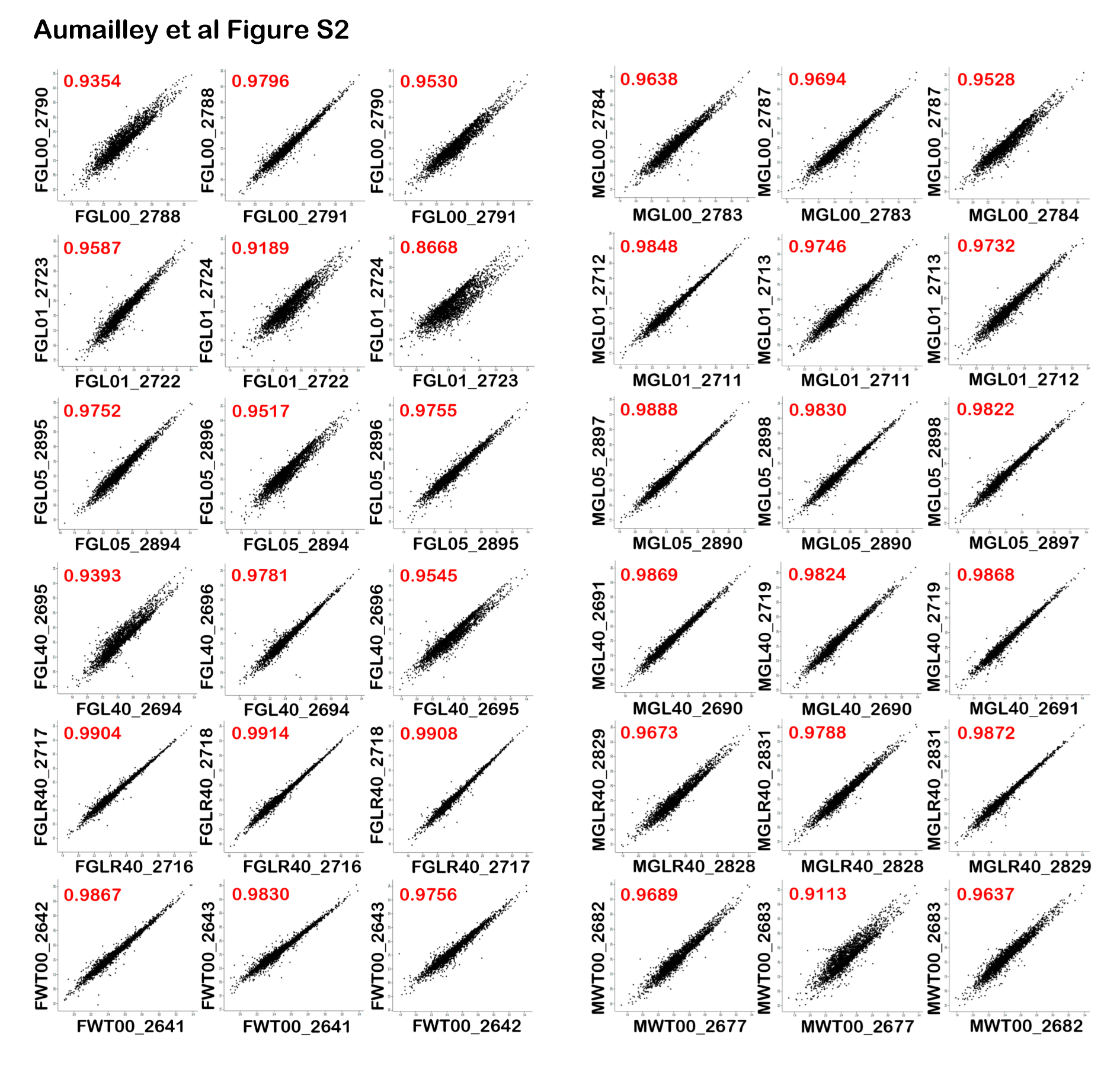

Supplement: Supplementary file 4 — Additional file 4: Figure S2 [file 40659_2024_509_MOESM4_ESM.png]

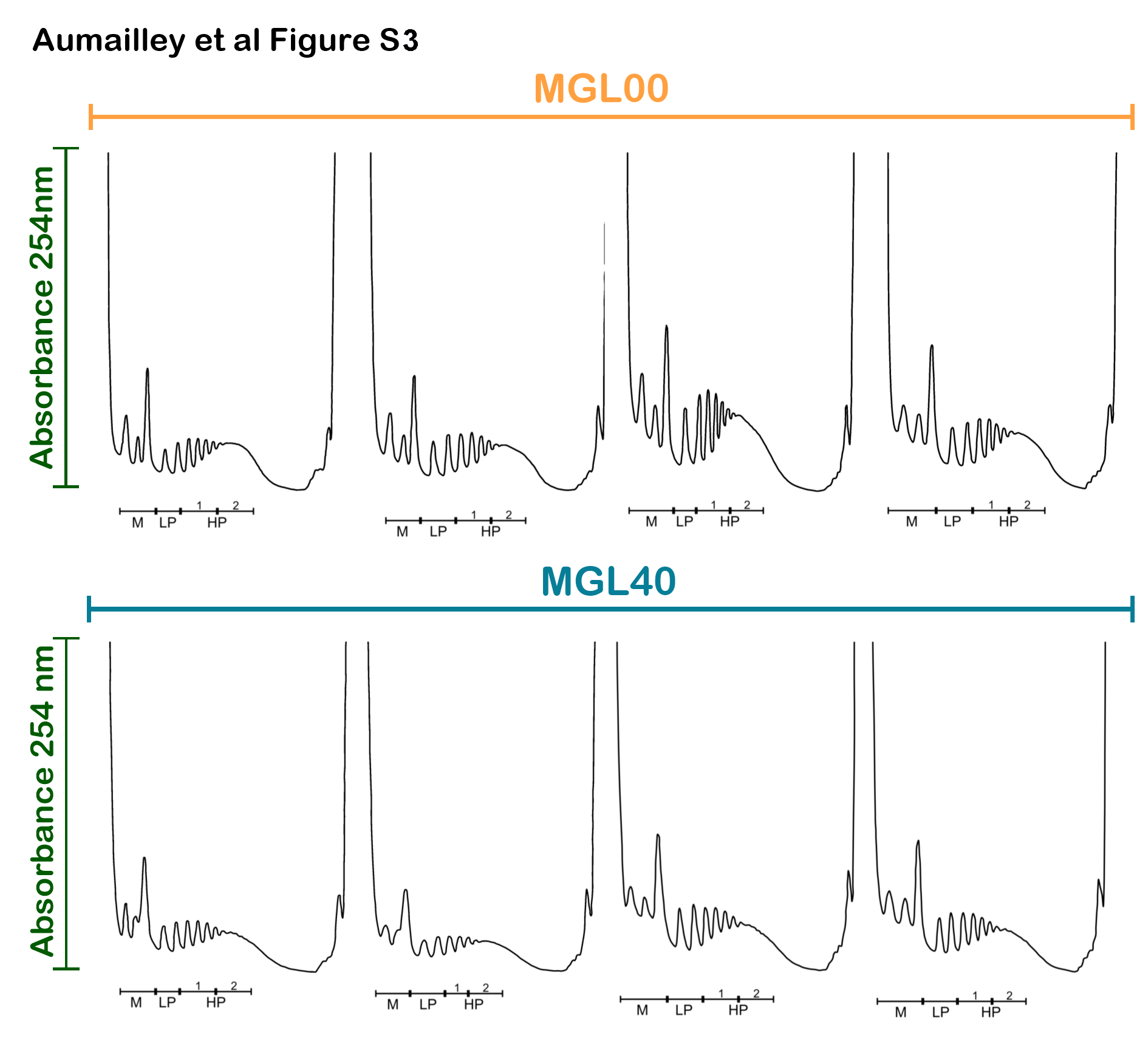

Supplement: Supplementary file 19 — Additional file 19: Figure S3 [file 40659_2024_509_MOESM19_ESM.png]

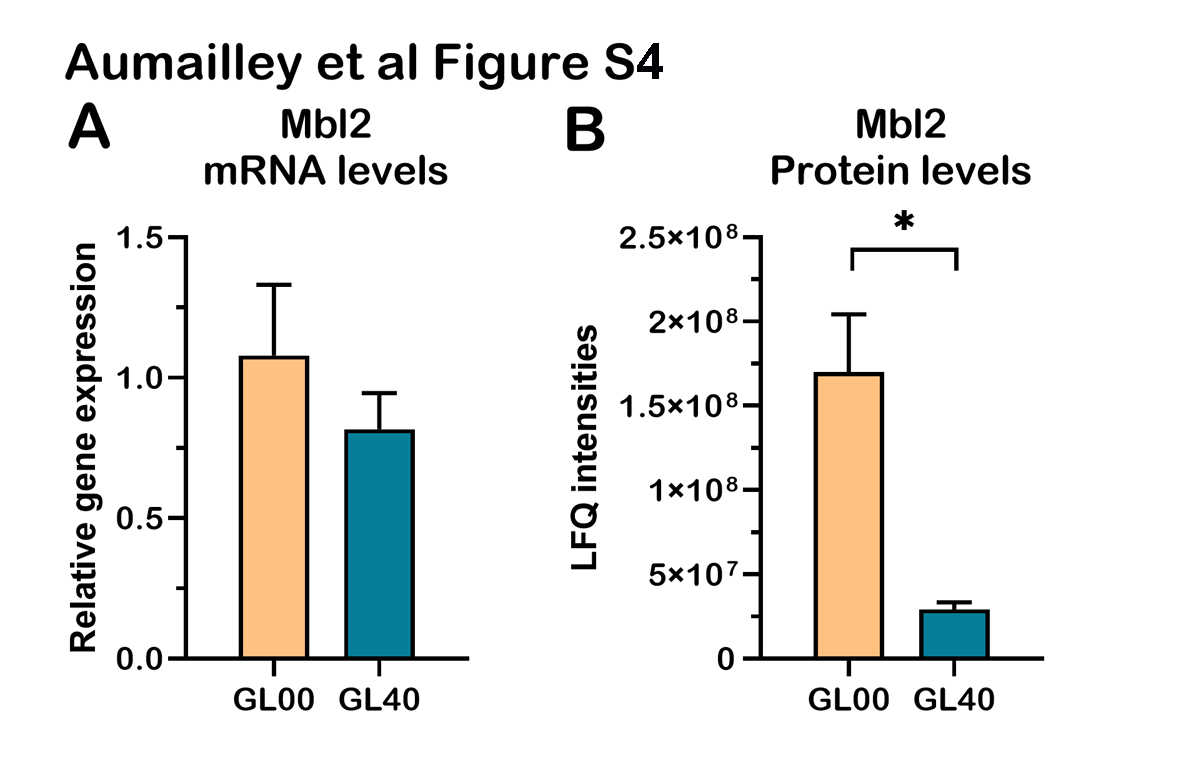

Supplement: Supplementary file 20 — Additional file 20: Figure S4 [file 40659_2024_509_MOESM20_ESM.png]
